# Beneficial soil microbiome profiles assembled using tetramycin to alleviate root rot disease in *Panax notoginseng*

**DOI:** 10.3389/fmicb.2025.1571684

**Published:** 2025-04-29

**Authors:** Lianjin Liu, Zongqing Wang, Cheng Luo, Yinglong Deng, Wentao Wu, Yongping Jin, Yuxuan Wang, Hongping Huang, Zhaoxia Wei, Youyong Zhu, Xiahong He, Liwei Guo

**Affiliations:** ^1^State Key Laboratory for Conservation and Utilization of Bio-Resources, Yunnan Agricultural University, Kunming, Yunnan, China; ^2^Key Laboratory of Agro-Biodiversity and Pest Management of Education Ministry of China, Yunnan Agricultural University, Kunming, Yunnan, China; ^3^Key Laboratory of Forest Resources Conservation and Utilization in the Southwest Mountains of China Ministry of Education, Southwest Forestry University, Kunming, Yunnan, China

**Keywords:** *Panax notoginseng*, root rot disease, tetramycin, differential microorganisms, key strains

## Abstract

**Background:**

Root rot disease is a major threat to the sustainable production of *Panax notoginseng*. Tetramycin has a broad-spectrum fungicidal efficacy, low toxicity, and high efficiency, However, the prevention and control of root rot disease of *P. notoginseng* and the specific mechanism of action are still unclear.

**Methods:**

In this paper, a combination of indoor and pot experiments was used to assess the effectiveness of tetramycin at alleviating root rot disease challenges encountered by *P. notoginseng*. Amplicon sequencing, metagenomic analysis with microbial verification were used to investigate the microecological mechanisms underlying tetramycin’s ability to reduce soil biological barriers.

**Results:**

We found that tetramycin significantly inhibited mycelial growth and spore germination of pathogenic fungi. Tetramycin, T2 (1000×) and T3 (500×), applied to continuous cropping soil, increased the seedling survival rates of *P. notoginseng*. Additionally, tetramycin reduced fungal α-diversity and shifted the fungal community assembly from deterministic to stochastic process. The microbial functions influenced by tetramycin were primarily associated with antibiotic synthesis and siderophore synthesis. Antibiotic efflux and inactivation have also been identified as the main resistance mechanisms. Microbial verification results showed that the artificially assembled tetramycin-regulated microbial community could indeed alleviate the occurrence of diseases. Furthermore, the cross-kingdom synthetic community assembled by the three key strains (*Pseudomonas aeruginosa, Variovorax boronicumulans*, and *Cladosporium cycadicola*) significantly improved the control of root rot disease and promoted plant growth.

**Discussion:**

This study provides novel insights into developing efficient biological control strategies and elucidates the role and mechanism of tetramycin in modulating soil microflora assembly to strengthen host disease resistance.

## Graphical Abstract

Conceptual model showing that tetramycin forms a rhizosphere barrier by reshaping the structure and function of microbial communities, demonstrating the potential mechanism of tetramycin rhizosphere inhibition of fungal pathogens. Created using BioRender.com (with permission). 
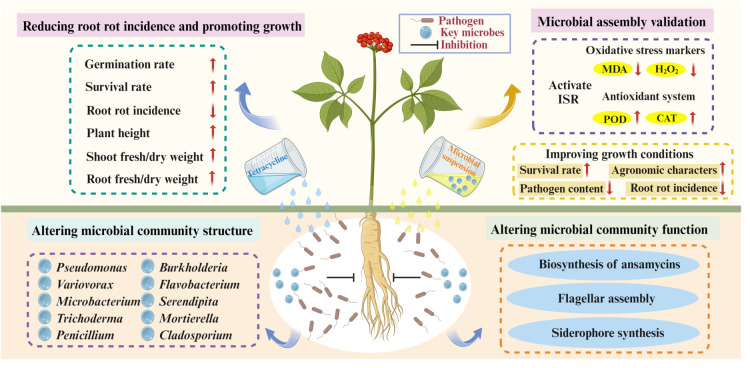

## 1 Introduction

Soil-borne diseases significantly constrain the healthy growth of crops. The abnormal accumulation of pathogens resulting from soil microbial imbalances represents a critical factor in the outbreak of these diseases (Chen et al., 2024). Effective management of soil-borne diseases is challenging due to the broad host range, mixed infections involving multiple pathogens, and the persistent survival of these pathogens in the soil (Nelson et al., 2018; Bischoff Nunes and Goodwin, 2022). Various strategies such as crop rotation, chemical application, and steam sterilization have been implemented to mitigate soil-borne diseases (Yang et al., 2019; Sun et al., 2022). However, these methods face several limitations. For instance, the application of chemical pesticides leads to pesticide residues and environmental pollution (Luo et al., 2021), while steam sterilization adversely affects beneficial microorganisms in the soil (Xu et al., 2016). In recent years, with rising concerns over food safety and human health, green prevention and control strategies have gained prominence as a viable approach to addressing these challenges.

The rhizosphere microbiome serves as the first line of defense against soil pathogens and is often referred to as the “second genome” of plants (Berendsen et al., 2012; Venturi and Keel, 2016). These microorganisms interact with plants through mutualistic relationships, playing a critical role in regulating nutrient absorption and enhancing plant immunity (Yuan et al., 2018). An increasing number of rhizosphere microorganisms are being identified for their potential as biocontrol agents and soil regulators (Koprivova and Kopriva, 2022). Furthermore, the application of fungicides has been shown to foster the assembly of disease-resistant microorganisms in the soil, thereby inhibiting pathogenic fungi and reducing the incidence of soil-borne diseases (Xie et al., 2023). These microorganisms enhance plant stress resistance through mechanisms such as competition, antibiosis, hyperparasitism, and induced systemic resistance (Pieterse et al., 2014; Yuan et al., 2018). Moreover, microbial natural products offer advantages such as novel structures, significant biological activity, and easy degradation, establishing themselves as a new frontier in agricultural biopharmaceutical research (Magrini et al., 2015). Notably, validamycin, wuyimycin, and tetramycin are typical bioactivators found in agricultural antibiotics and are widely utilized worldwide (Dayan et al., 2009; Xie et al., 2023).

Tetramycin, also known as wuningmycin, is a 26-membered tetraene macrolide antibiotic produced by the non-adsorbing strain Streptomyces sylvestris subsp. It consists of four integral components: A1, A2, B, and C. Among them, A1 and A2 are classified as macrolides and tetraphenyl antibiotics, while B is a peptide pyrimidine nucleotide antibiotic, and C is a nitrogen-containing heterocyclic compound (Ren et al., 2014). Tetramycin is effective for controlling several fungal diseases, including head blight caused by *Fusarium* and rice blast caused by *Magnaporthe grisea* (Shi et al., 2020; Zhao et al., 2010). Tetramycin can improve the soil micro-ecological environment and significantly reduce the richness and diversity of fungi, thereby controlling fungal populations and mitigating disease (Wei, 2021; Wu et al., 2022). Overall, the impact of tetramycin on the structure of soil fungal microbial communities is markedly greater than its effect on bacteria. Compared with conventional chemical treatments, tetramycin is a broad-spectrum, low-toxicity, efficient, and environmentally friendly microbial fungicide.

Following infection by root rot pathogens, *P. notoginseng* undergoes root decay, leaf chlorosis and wilting, and stem blight, which can result in yield reductions ranging from 5% to 20%; in severe cases, reductions can exceed 70%. Such losses constitute approximately 70%–85% of the total yield losses. It is a key factor restricting the sustainable development of the *P. notoginseng* cultivation industry and is a major contributor to the challenges associated with continuous cropping. The alteration of soil microbial communities is one of the primary factors leading to root rot disease in *P. notoginseng*. Research has demonstrated that the obstacles associated with continuous cropping, resulting from root rot disease, are closely linked to the downregulation of rhizosphere microbial diversity, structural imbalances, and the degradation of micro-ecological environments (Liu et al., 2018; Zhang et al., 2019). For instance, after several years of planting *P. notoginseng*, there is a marked increase in soil-borne pathogens, such as *F. oxysporum* and *F. solani* of the phylum Ascomycota, accompanied by a decrease in the abundance of beneficial bacterial communities (Liu et al., 2018; Luo et al., 2019). Among them, soil microbial imbalance is the key factor causing *P. notoginseng* root rot disease. Nonetheless, the challenges associated with using tetramycin to alleviate root rot disease in *P. notoginseng* cultivation have not been documented.

Given the increasing public concern about the environment, we used *P. notoginseng* as a model plant to answer four main questions: (1) Does tetramycin inhibit the mycelial growth and spore germination of the main pathogens of *P. notoginseng* root rot disease *in vitro*? (2) Can the application of tetramycin in *P. notoginseng* continuous cropping system effectively alleviate the occurrence of root rot disease? (3) What is the correlation between tetramycin and rhizosphere microorganisms that mediate the inhibition of soil-borne diseases? (4) Whether the artificially assembled tetramycin-regulated differential microorganisms can resist the stress of root rot pathogens? To address these questions, we first carried out indoor virulence determination. Subsequently, we carried out pot experiments and omics analysis. Finally, we studied the individual and combined effects of differential microorganisms regulated by tetramycin on root rot disease control, plant growth promotion, and host plant-induced systemic resistance (ISR) activation. Overall, this study aimed to elucidate the effect and mechanism of tetramycin on reducing the occurrence of *P. notoginseng* root rot disease and provide valuable data for guiding sustainable soil management and agroecosystems.

## 2 Materials and methods

### 2.1 Effect of tetramycin on mycelial growth of root rot pathogens of *P. notoginseng*

Three pathogenic strains-*Fusarium oxysporum*, *Fusarium solani*, and *Cylindrocarpon destructans* (obtained from Yunnan Agricultural University)-were utilized to evaluate the effects of tetramycin on mycelial growth. The effect of tetramycin on mycelial growth was evaluated following the method described by Ma et al. (2018), with concentrations established based on the study by Gao et al. (2018): Juvenile hyphae (5 mm) excised from the active margins of 7-day-old colonies and inoculated onto PDA plates with a range of tetramycin concentrations: 0, 0.3, 0.6, 0.9, 1.2, and 1.5 mg/L, PDA medium without tetramycin serving as the control, The plates were incubated at 25°C for 7 days under a 12 h dark/light cycle. The radial mycelial growth of all profiles was measured using the crisscross method. The experiment was repeated at least twice with four replicates for each treatment. The inhibition ratio of mycelial growth were calculated using Equation 1:


(1) Inhibition⁢rate⁢of⁢mycelial⁢growth=[(Xc-Xi)/Xc]×100%

where Xc is the mean colony radius of the control media, and Xi is the colony radius of the tetramycin-amended media.

### 2.2 Effect of tetramycin on spore germination of root rot pathogens of *P. notoginseng*

Spore suspensions of the three pathogenic strains were prepared using sterile distilled water to achieve a final concentration of 1.0 × 10^6^ CFU/mL. Subsequently, 100 μL of each spore suspension was spread onto the surface of agar plates containing tetramycin at concentrations of 0.0042, 0.005, 0.0062, 0.0083, 0.0125, and 0.0251 mg/L, with agar plates without tetramycin serving as the control, the plates were then incubated at 25°C in complete darkness for 12 h. The number of spores (germinated and non-germinated) was counted under a light microscope (BX43, Olympus). The spore was deemed to have germinated when the length of its germinal tube had reached a length equivalent to half of the spore diameter (Plascencia-Jatomea et al., 2003). Three replicates were used for each treatment, and each repetition was observed for 3 visual fields. According to the statistical level of each visual field, spore germination percentages and the inhibition ratio of spore germination were calculated using Equations 2, 3:


(2) Sporegermination=Numberofgerminatedspores/

Total⁢number⁢of⁢spores⁢investigated×100%

(3) Inhibition⁢ratio⁢of⁢spore⁢germination=[(Nc-Ni)/Ni]×100%

Where Nc is the spore germination ratio of the control media, and Ni is the spore germination ratio of the tetramycin-amended media.

### 2.3 Experimental design and evaluation of tetramycin effects on *P. notoginseng* growth

The pot experiment was carried out at the greenhouse of Yunnan Agricultural University experimental station in Xundian county, Yunnan province, China. The soil that was continuously planted and not treated was collected from *P. notoginseng* planted for 3 years and sub-packed in a flowerpot (60 cm × 40 cm × 15 cm). In the first year, we diluted tetramycin into T1 (2000×), T2 (1000×), and T3 (500×) concentrations in December. All treatments were irrigated with 4 L tetramycin once a day for 3 consecutive days, equal volume of distilled water was irrigated as control (T0). One week later, healthy *P. notoginseng* seeds (104 seeds) were planted in each pot. To ensure the accuracy of the experiment, we repeated it in the second year and divided the three-year continuous cropping soil into two subsamples: one was not treated and the other was autoclaved. Based on the concentration setting of the first year, T4 (250×) concentration was added, and the 15–30 cm soil layer of Pinus tabulaeformis forest in Xundian county was considered as the new soil control (TX). Each treatment was repeated four times using a randomized block design. All pots were placed in the greenhouse to allow 10% light transmission, and the temperature was maintained at < 30°C to help facilitate *P. notoginseng* growth (Wang et al., 2020). To evaluate the effects of tetramycin on the growth of *P. notoginseng*, seed germination was investigated in each pot in April of the next year. Furthermore, the survival rate, plant height, fresh and dry biomass were measured in July.

### 2.4 Soil sampling

A previously described method with minor modifications was followed to collect soil samples (Wang et al., 2020). Briefly, during the harvest period in September, rhizosphere soil samples under the last repeated experiment were collected. The collection method is to gently remove the *P. notoginseng* plants from the soil. The plant roots were shaken vigorously to separate loosely attached soil. The tightly attached soil was brushed off and passed through a 2-mm grid to remove debris as rhizosphere soil. The soil was divided into two parts. One sample was stored at −4°C for the isolation of soil-culturable microorganisms. Another sample was stored at −80°C for microbial community analysis.

### 2.5 Rhizosphere microbiome analysis

Based on a 2-year pot experiment, we selected rhizosphere soils under T0 (0), T2 (1000×), and T3 (500×) treatments for microbial community studies. Total genomic DNA from the above rhizosphere soils was extracted using a DNeasy^®^ PowerSoil^®^ Pro Kit (MO BIO Laboratories, Inc., Carlsbad, CA, USA). DNA purity and concentration were determined using a NanoDrop2000TM spectrophotometer. ITS region of ribosomal DNA was amplified with primers ITS1F (5′-CTTGGTCATTTAGAGGAAGTAA-3′) and ITS4R (5′-GCTGCGTTCTTCATCGATGC-3′). Bacterial genes in the V3–V5 region of the 16S rRNA gene were amplified with primers 338F (5′-ACTCCTACGGGAGGCAGCAG-3′) and 806R (5′-GGACTACHVGGGTWTCTAAT-3′) (Zeng and An, 2021). PCR products were purified and qualified using the manufacturer’s protocol. Purified amplicons were pooled and sequenced using the Illumina MiSeq PE300 platform.

The sequenced raw reads were demultiplexed, quality-filtered using fastp version 0.20.0 (Chen et al., 2018) and merged using FLASH version 1.2.7 (Magoč and Salzberg, 2011). Operational taxonomic units (OTUs) with a 97% similarity cutoff (Stackebrandt and Goebel, 1994; Edgar, 2013) were clustered using UPARSE version 7.1 (Edgar, 2013), and chimeric sequences were identified and removed. The taxonomy of each OTU representative sequence was analyzed using the RDP Classifier version 2.2 (Wang et al., 2007) against the 16S rRNA database (Silva v138) and ITS database (Unite v 8.0), with a confidence threshold of 0.7.

### 2.6 Metagenomic sequencing and analysis

Consistent with the samples used for microbiome sequencing, the same method was used to extract DNA. The DNA was fragmented using a Covaris M220, resulting in fragments of approximately 400 bp. A paired-end library was constructed using the NEXTFLEX Rapid DNA-Seq Kit, followed by metagenomic sequencing on an Illumina NovaSeq platform. The raw data were quality-controlled using Fastp. BWA software aligned reads with the host DNA sequence. Contaminated and similar reads were excluded. The MEGAHIT software was used to assemble the sequences. The final assembly comprised contigs of at least 300 bp. ORFs of the assembled contigs were predicted using MetaGene. CD-HIT was used to cluster the predicted gene sequences, creating a non-redundant set. High-quality reads were compared with the non-redundant set (95% identity) using SOAPaligner, enabling quantification of gene abundance. BLASTP was used to compare the amino acid sequences of the non-redundant gene set with the KEGG genes database and the CARD database, providing information regarding KEGG functions and antibiotic resistance annotations corresponding to the genes. All sequencing services mentioned above were performed by Shanghai Majorbio Bio-pharm Technology Co., Ltd.

### 2.7 Isolation of differential microorganisms from soil treated with tetramycin

Differential microorganisms were isolated following Haiyambo et al.’s (2015) method. Genomic DNA of all microorganisms was extracted using the Aidelan Genomic DNA Rapid Extraction Kit (Beijing, China), and universal primers for 16S rDNA or ITS were used for PCR amplification. The amplified products were sequenced by Beijing Tsingke Biotech Co., Ltd. The resulting sequences were subjected to sequence homology alignment using the NCBI website to ascertain their homology with other sequences in the database. The antagonistic effect of the strain on the pathogen was determined by confrontation culture. The isolated differential microorganisms were stored in a solution of 30% glycerol at a temperature of −80°C.

### 2.8 Functional detection of strains

The siderophore-producing capability of the strain was assessed using a CAS plate assay. 6 μL of the bacterial suspension was inoculated onto the CAS detection plate (fungal inoculation with 5 mm diameter juvenile hyphae). Following 72 h of incubation at 28°C, the color changes in the medium surrounding the colonies were observed (Peng et al., 2020). Additionally, the isolated bacteria were inoculated onto a nitrogen-free medium (fungal inoculation with 5 mm diameter juvenile hyphae) and incubated at 28°C for 72 h. The growth of the colonies was monitored to evaluate the nitrogen-fixing ability of the strains (Dai et al., 2017).

### 2.9 Differential microorganisms and assembly verification

The new soil was autoclaved and sub-packed in a plastic flowerpot and the fresh *P. notoginseng* seedlings were transplanted into the flowerpot, and the seedlings were slowed for 7 days. The concentration of the differential bacterial suspension was OD_600_ = 0.6. In addition, the spore suspension of *F. solani* or differential fungi was collected and the number of spores was adjusted to 10^7^ CFU/mL.

The effects of these differential microorganisms on the prevention and control of root rot disease and the promotion of growth in *P. notoginseng* were investigated through individual strain treatments and the construction of various combinations of synthetic communities (SynComs). For the bacterial disease prevention verification, the treatments included: five single bacterial treatments (BS1, BS2, BS3, BS4, BS5), antagonistic bacterial combination (BSMC1), non-antagonistic bacterial combination (BSMC2), and combination of both antagonistic and non-antagonistic bacteria (BSMC3). For fungal disease prevention verification, the treatments consisted of five single strain treatments (FS1, FS2, FS3, FS4, and FS5), high antagonistic fungal combination (FSMC1), low antagonistic fungal combination (FSMC2), and combination of high and low antagonistic fungal (FSMC3). Additionally, to assess the function of key microorganisms, two specific bacteria were tested in combination (BFSMC1), and two key bacteria were combined with one key fungus (BFSMC2). In this experiment, add only *F. solani* (P) or water (W) were used as controls. The specific composition of each treatment is detailed in Supplementary Table 1.

Potted *P. notoginseng* was irrigated with 100 mL of the aforementioned different microbial suspension, while SynComs were applied by mixing in equal volumes for irrigation. After 24 h, the plants were irrigated with an equal volume of pathogen spore suspension. Moreover, a treatment involving only the irrigation of different microorganisms without adding pathogens was implemented to explore the corresponding growth-promoting effects. This irrigation process was performed once a month for a total of two times. A randomized block design was used for each treatment, with six replicates. Seven days after irrigation, samples of *P. notoginseng* from different treatments were collected, and the resistance levels of the plants were evaluated by measuring root enzyme activity. After cultivating *P. notoginseng* for three months and subsequently harvesting, assessments were conducted to determine the survival rate, disease incidence, and agronomic traits of the plants. Additionally, rhizosphere soil samples were collected and the pathogen content was quantified using qPCR.

### 2.10 qPCR of *F. solani*

The *F. solani* content in the rhizosphere was quantitatively analyzed using fluorescence quantitative PCR. The FastBeat Soil DNA Extraction Kit (Aidlab, Beijing) was used for the extraction of total soil DNA, and specific primers were used for the quantitative detection of the target sequence: forward primers Fs-QF (5′-CCACGCTTGTGAGCTATGTAGTTC-3′) and reverse primers Fs-QR (5′-CTCTCTTGAGGTAGACCACAGTAGGC-3′). The reaction system was prepared with a total volume of 18.4 μL, comprising 7 μL of nuclease-free water, 10 μL of 2 × SYBR Green Mix, 0.2 μL of Fs-QF, 0.2 μL of Fs-QR, and 1 μL of DNA template. The reaction procedure commenced with a 2-min incubation at 95°C, followed by 45 cycles. Each cycle comprised 1 min at 95°C, 30 s at 62°C, and 1 min at 72°C, with fluorescence measured at 72°C. The DNA templates were used in four replicates (Li, 2020).

### 2.11 Induced systemic resistance assay

The roots of *P. notoginseng* under each treatment were collected. Subsequently, the contents of peroxidase (POD), catalase (CAT), malondialdehyde (MDA), and hydrogen peroxide (H_2_O_2_) were measured using kits (Grace, Suzhou).

### 2.12 Statistical analyses

The DPS v9.5 was used to calculate the virulence regression equation and EC_50_ value, while the “agricolae” package in R 4.2.3 was used for variance analysis. Principal coordinate analysis (PCoA) based on Bray-curtis distance was performed using a “vegan” package. The impact of soil types and tetramycin on community dissimilarities was evaluated using PERMANOVA, and Pearson method was used to evaluate the relationship between tetramycin and microbial diversity. The results were analyzed using ggplot2. OTUs with abundances > 0.1% were selected for this analysis of the community construction process. The βNT1 value was calculated using the comdist equation from the “phylocom” package. The βNTI value was defined as deterministic process (|βNTI| ≥ 2) or stochastic process (|βNTI| < 2). Furthermore, deterministic and stochastic processes were classified into five ecological processes based on βNTI and RC_Bray_ values. They included homogeneous selection (βNTI < −2), variable selection (βNTI > +2), dispersal limitation (|βNTI| < 2 and RC_Bray_ > 0.95), homogenizing dispersal (|βNTI| < 2 and RCBray < –0.95), and undominated (|βNTI| < 2 and |RC_Bray_| < 0.95) (Jiao et al., 2020). Use the DESeq2 package to calculate the fold change (FC) and *p*-values for each microbial genus, and *p*-values were adjusted using the False Discovery Rate (FDR). Set criteria of |FC| > 2 and *p* < 0.05 to analyze the patterns of microbial changes. The Generalized Reporter Score-based Analysis (GRSA) method was used to evaluate alterations in the KEGG pathway^1^ (Peng et al., 2024). Spearman correlation scores (|*r*| > 0.9; *p* < 0.01) were used for visual network analysis with Cytoscape v. 3.6.0. A neighbor-joining phylogenetic tree of the isolated microorganisms was constructed using MEGA 11 software.

## 3 Results

### 3.1 Tetramycin reduces root rot disease by inhibiting the growth of pathogens

The mycelial growth of the three target pathogens was significantly inhibited by treatments with various concentrations of tetramycin (Supplementary Figure 1). The EC_50_ values for mycelial growth were 0.60, 1.52, and 0.52 mg/L, respectively (Supplementary Table 2). Furthermore, the spore germination rate of *F. oxysporum*, *F. solani*, and *C. destructans* progressively declined with increasing concentrations of tetramycin (Supplementary Figure 2), with EC_50_ values of 0.0064, 0.0122, and 0.008 mg/L, respectively (Supplementary Table 2).

The verification results from the first year indicated that the seedling emergence rates for all treatments were higher than that of the clear water control (T0) following tetramycin treatment. Specifically, the emergence rates for the T2 and T3 treatments were 179.96% and 212.18% higher than that of T0, respectively, while the emergence rate for the T1 treatment was 51.09% higher than that for T0 (Supplementary Figure 3A). Additionally, the seedling survival investigation revealed that the survival rates for T2 and T3 increased by 432.52% and 530.03%, respectively, compared with that of T0 (Supplementary Figure 3B). Furthermore, the root rot incidence was significantly reduced following the T2 and T3 treatments (Supplementary Figure 3C).

**FIGURE 1:**
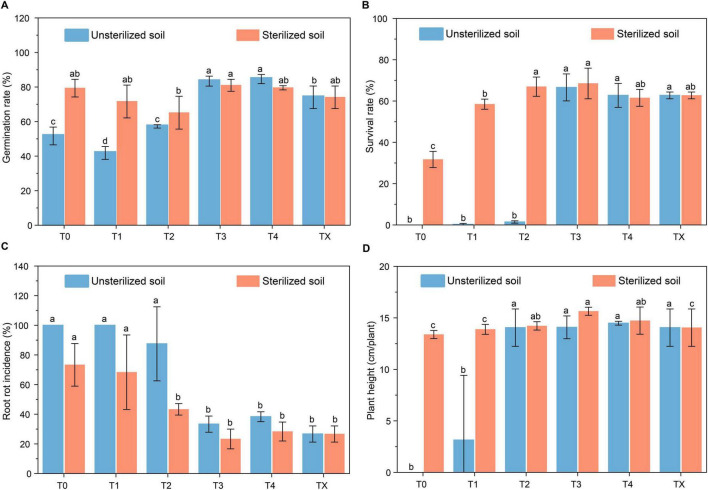
Effect of tetramycin on alleviating root rot disease of *P. notoginseng*. **(A)** Effects of tetramycin on the germination rate. **(B)** Effects of tetramycin on the survival rate. **(C)** Effects of tetramycin on the root rot incidence. **(D)** Effects of tetramycin on the plant height. Different lowercase letters indicate significant differences within groups, *p* < 0.05. The data in the figure are the mean ± standard deviation of four biological replicates. The *p*-values were calculated using a one-way analysis of variance and multiple comparisons. T0: water; T1: tetramycin 2000×; T2: 1000×; T3: tetramycin 500×; T4: tetramycin 250×; TX: new soil under the forest.

The verification results in the second year showed that tetramycin had a beneficial effect on promoting the growth of *P. notoginseng* and reducing the root rot incidence in unsterilized soil. Compared with that under T0 (51.68%), the emergence rate of *P. notoginseng* under T4 treatment significantly increased by 63.74% (Figure 1A). The survival rate improved markedly from complete mortality at T0 to 66.59% after T3 (Figure 1B). Furthermore, the root rot incidence significantly decreased from 100% at T0 to 66.67% at T3 (Figure 1C). Notably, plant height was the highest (14.48 cm/plant) in the T4 treatment (Figure 1D). Analysis of the agronomic traits showed that they were absent from T0 because there was no survival. The highest biomass growth was observed in T3. Specifically, the shoot fresh and dry weights increased to 0.80 g/plant and 0.36 g/plant (Supplementary Figures 4A, B). Conversely, the root fresh and dry weights increased to 0.88 g/plant and 0.64 g/plant (Supplementary Figures 4C, D). In the sterilized soil, the germination (Figure 1A) and survival rate (Figure 1B) improved by 2.12% and 115.91%, respectively, whereas the root rot incidence decreased by 68.18% after T3 (Figure 1C). The observed changes in agronomic traits followed a fluctuating pattern similar to that in unsterilized soil (Supplementary Figures 4A, B).

### 3.2 Altering the diversity levels and community composition of root-associated microorganisms

**FIGURE 2:**
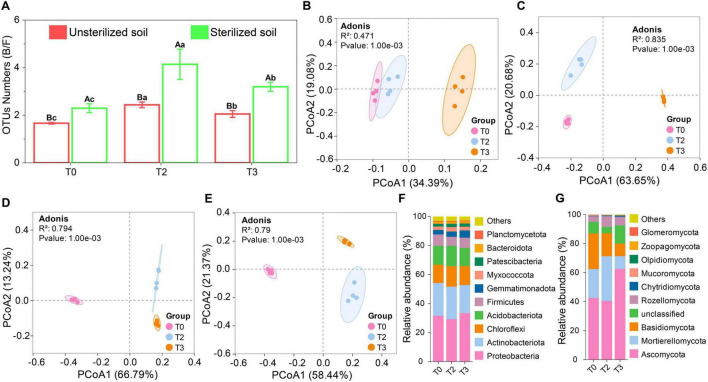
Effects of tetramycin on the structure and composition of rhizosphere microbial community. **(A)** Proportion of OTUs (B/F). **(B)** Bacterial PcoA analysis based on OTU level in unsterilized soil. **(C)** Fungal PcoA analyses based on the OTU level in unsterilized soil. **(D)** Bacterial PcoA analysis based on OTU level in sterilized soil. **(E)** Fungal PcoA analyses based on the OTU level in sterilized soil. **(F)** Bacterial species composition analysis based on phylum level in unsterilized soil. **(G)** Fungal Species composition analysis based on phylum level in unsterilized soil. Different lowercase letters indicate significant differences within groups, *p* < 0.05. The data in the figure are the mean ± standard deviation of four biological replicates. The *p*-values were calculated using a one-way analysis of variance and multiple comparisons. T0: water; T2: 1000×; T3: tetramycin 500×.

Microbial sequencing revealed that the rhizosphere contained 7,754 bacterial and 5,280 fungal OTUs. The application of tetramycin significantly increased the bacterial-to-fungal (B/F) OTU ratio (Figure 2A). PCoA demonstrated substantial differences in the bacterial and fungal communities in unsterilized and sterilized soils treated with tetramycin (Figures 2B, C). The results of alpha diversity assessments indicated no significant effect of bacteria following tetramycin treatment in unsterilized soil (Supplementary Figures 5A, B). However, fungal alpha diversity declined significantly (Supplementary Figures 5C, D). In sterilized soil, tetramycin significantly increased bacterial diversity (Supplementary Figures 5A, B) while decreasing fungal alpha diversity (Supplementary Figures 5C, D). Correlation analyses revealed a significant negative correlation between the Chao index (*r* = −0.65, *p* < 0.05) and Shannon index (*r* = −0.82, *p* < 0.01) of fungi in unsterilized soil and tetramycin (Supplementary Figure 5E). Additionally, the Shannon index of bacteria (*r* = 0.88, *p* < 0.01) and fungi (*r* = −0.87, *p* < 0.01) in sterilized soil showed a significant correlation (Supplementary Figure 5F).

The analysis of the microbial community at the top 10 phylum levels revealed that in unsterilized soil, tetramycin treatment had minimal impact on the bacterial phylum composition, with Proteobacteria and Actinobacteriota being the dominant bacterial phyla (Figure 2D). The abundance of Gemmatimonadota under T2 and T3 treatments increased by 15.53% and 59.74%, respectively, compared to T0. In the fungal community, the dominant phyla were Ascomycota and Mortierellomycota (Figure 2E); although the overall abundance of the fungal community decreased following tetramycin treatment, Ascomycota abundance under T3 treatment increased by 46.69% compared with T0. In sterilized soil, the dominant bacterial phylum shifted from Actinobacteriota in T0 to Proteobacteria in T2 and T3 (Supplementary Figure 6A), with the abundance of Proteobacteria increasing by 15.95% and 25.26%, respectively. For fungi, Ascomycota remained the dominant phylum in the fungal community (Supplementary Figure 6B), with its abundance under T2 and T3 treatments increasing by 27.62% and 44.57%, respectively, compared with that under T0.

### 3.3 Recruiting differential microorganisms and key microorganisms

**FIGURE 3:**
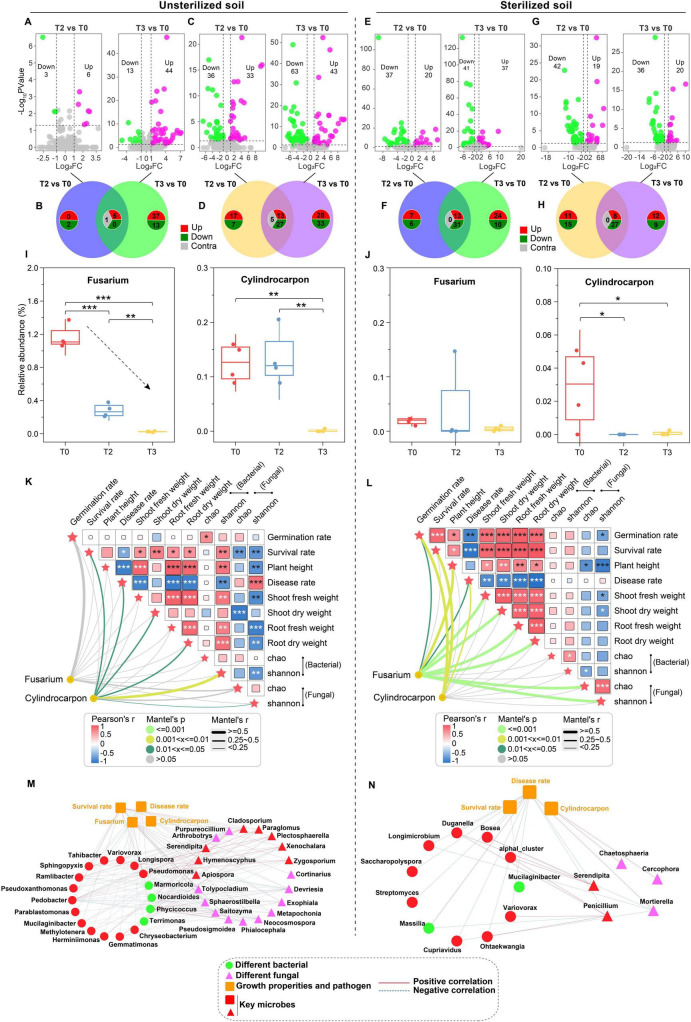
Difference analysis based on genus level. **(A)** Differential bacteria in unsterilized soil. **(B)** Number of unique differential bacteria in unsterilized soil. **(C)** Differential fungal in unsterilized soil. **(D)** Number of unique differential fungi in unsterilized soil. **(E)** Differential bacteria in sterilized soil. **(F)** Number of unique differential bacteria in sterilized soil. **(G)** Differential fungal in sterilized soil. **(H)** Number of unique differential fungi in sterilized soil. **(I)** Abundance distribution of *Fusarium*. **(J)** Abundance distribution of *Cylindrocarpon*. **(K)** Mantel analysis of pathogen genus and key indicators in unsterilized soil. **(L)** Mantel analysis of pathogen genus and key indicators in sterilized soil. **(M)** Correlation analysis between differential microorganisms and key indicators in unsterilized soil. **(N)** Correlation analysis between differential microorganisms and key indicators in sterilized soil. The significant correlations are presented as asterisks (*P < 0.05; **P < 0.01; ***P < 0.001). The data in the figure are the mean ± standard deviation of 4 biological replicates The *p*-values were calculated using one-way analysis of variance and multiple comparisons. T0: water; T2: 1000×; T3: tetramycin 500×.

The differential analysis at the genus level indicated that the number of upregulated bacterial genera increased from 6 in T2 to 44 in T3 in unsterilized soil (Figure 3A). The unique upregulated bacterial genera rose from 0 in T2 to 37 in T3, with 6 of these genera being up-regulated concurrently (Figure 3B). The count of down-regulated fungal genera increased from 36 in T2 to 63 in T3 (Figure 3C), while the number of specific downregulated fungal genera increased from 7 to 33, with 27 of these genera being downregulated (Figure 3D). In sterilized soil, the number of upregulated bacterial genera rose from 20 in T2 to 37 in T3 (Figure 3E). The unique upregulated bacterial genera increased from 7 in T2 to 24 in T3, 13 of which were co-upregulated (Figure 3F). The count of downregulated fungal genera decreased from 42 in T2 to 36 in T3 (Figure 3G), while the number of specific down-regulated fungal genera declined from 15 in T2 to 9 in T3 (Figure 3H).

We analyzed the abundance of rhizosphere pathogens and found that in unsterilized soil, compared with T0, T2 and T3 treatments significantly reduced the abundance of *Fusarium* by 75.91% and 97.82%, respectively, with T3 treatment also significantly decreasing the abundance of *Cylindrocarpon* by 98.99% (Figure 3I). In sterilized soil, both T2 and T3 treatments significantly lowered the abundance of *Cylindrocarpon* (Figure 3J). Mantel analysis indicated that *Fusarium* had a greater impact on the growth and fungal diversity of *P. notoginseng* in unsterilized soil (Figure 3K), whereas *Cylindrocarpon* was primarily affected in sterilized soil (Figure 3L).

We also examined the relationships between the microorganisms and seedling survival rate, root rot incidence, and pathogen abundance. In unsterilized soil, 52 genera (bacteria: 23; fungi: 29) exhibited strong interactions (|*R*| > 0.9 and *p* < 0.01). Overall, 22 genera (bacteria: 14; fungi: 8) were identified as key genera (with survival rate *R* > 0.9 and other *R* < −0.9, *p* < 0.01) (Figure 3M). In sterilized soil, 16 genera (bacteria: 11; fungi: 5) displayed strong interactions (|*R*| > 0.9 and *p* < 0.01). Among these, 11 genera (bacteria: 9; fungi: 2) were classified as key genera (with survival rate *R* > 0.9 and other *R* < −0.9, *p* < 0.01) (Figure 3N).

### 3.4 Rhizosphere microbial community assembly process

**FIGURE 4:**
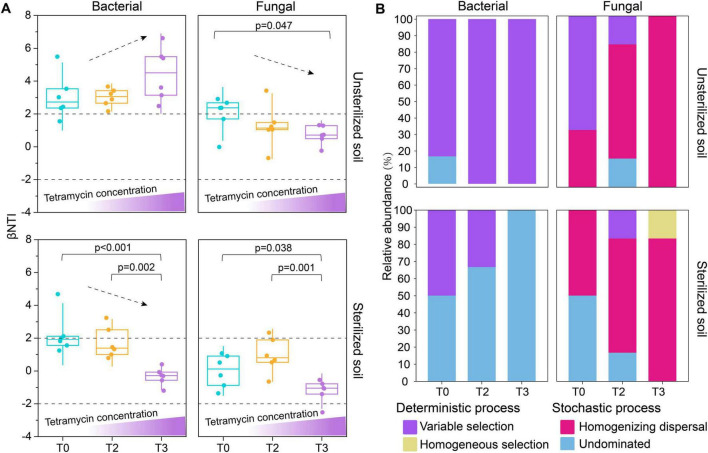
Effects of tetramycin on rhizosphere community assembly. **(A)** Contributions of deterministic and stochastic processes in rhizosphere community assembly. **(B)** Basic ecological processes involved in the assembly of rhizosphere communities. The significant differences (marked with *P*-value) of βNTI values between different treatments are listed above the boxplots. The *p*-values were calculated using a one-way analysis of variance and multiple comparisons. T0: water; T2: tetramycin 1000×; T3: tetramycin 500×.

The results of microbiome assembly based on zero model analysis revealed that the assembly of rhizosphere bacterial communities was predominantly influenced by deterministic processes (|βNTI| > 2) in unsterilized soil (Figure 4A). Additionally, the rhizosphere fungal community transitioned from deterministic process (|βNTI| > 2) in T0 to stochastic process (|βNTI| < 2) under T2 and T3 treatments (Figure 4A). Variable selection (βNTI > +2) dictated the deterministic processes involved in bacterial community assembly (Figure 4B). The mechanism driving fungal community assembly shifted from variable selection (βNTI > +2) in T0 to homogenizing dispersal (|βNTI| < 2 and RC_Bray_ < −0.95) in T2 and T3 (Figure 4B). In sterilized soil, the assembly of rhizosphere bacterial communities under T2 and T3 treatments was almost entirely governed by stochastic processes (|βNTI| < 2) (Figure 4A), while the assembly of the rhizosphere fungal community was dominated by stochastic processes (|βNTI| < 2) (Figure 4A). The assembly contribution of rhizosphere bacteria is mainly undominated (|βNTI| < 2 and |RC_Bray_| < 0.95) (Figure 4B). Furthermore, the contribution of stochastic—primarily driven by homogenizing dispersal (|βNTI| < 2 and RC_Bray_ < −0.95)—increased progressively from T0 to T2 and T3 (Figure 4B).

### 3.5 Functional analysis of the rhizosphere community

**FIGURE 5:**
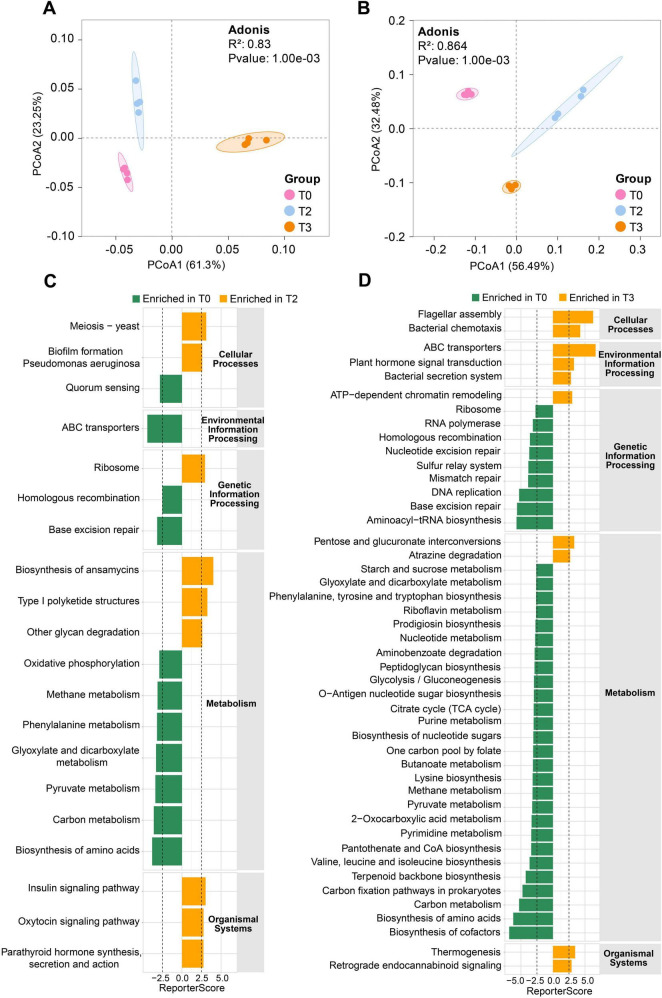
Generalized reporter score-based (GRSA) enrichment analysis. **(A)** PcoA analysis based on species level in unsterilized soil. **(B)** PcoA analysis based on species level in sterilized soil. **(C)** KEGG pathway enrichment analysis under T2 treatment in unsterilized soil. **(D)** KEGG pathway enrichment analysis under T3 treatment in unsterilized soil. The score threshold of the report was 2.5, and the corresponding confidence level was approximately 0.995. The human disease pathway was removed, and the significantly enriched pathways in T0 are displayed in green. The significantly enriched pathways in T2 or T3 are shown in orange. The data in the figure are four biological repetitions. T0: water; T2: 1000×; T3: tetramycin 500×.

Species-based PCoA revealed significant separation of microbial communities in both unsterilized and sterilized soils treated with tetramycin (Figures 5A, B). Metagenomic sequencing generated 9,741 KO functional genes. The GRSA analysis based on KO genes revealed that under unsterilized soil, the biofilm formation in *Pseudomonas aeruginosa* and biosynthesis of ansamycins pathways were significantly enriched under T2 treatment (Figure 5C). Important functions such as flagellar assembly, bacterial chemotaxis, ABC transporters, and bacterial secretion systems were significantly enriched under T3 treatment (Figure 5D). Genes involved in the flagellar assembly pathway, such as flagellar M-ring protein (*fliF*) and flagellar biosynthesis protein (flhA), along with key genes related to the bacterial chemotaxis pathway, including chemotaxis proteins (*cheZ, motC, motD*), were upregulated. In unsterilized soil, pathways involving flagellar assembly, bacterial chemotaxis, biosynthesis of siderophore group nonribosomal peptides, biosynthesis of enediyne antibiotics, and biosynthesis of ansamycins were significantly enriched under T2 treatment (Supplementary Figure 7A). Additionally, important pathways such as ABC transporters, biosynthesis of siderophore group nonribosomal peptides, biosynthesis of ansamycins, and tetracycline biosynthesis were significantly enriched under T3 treatment (Supplementary Figure 7B). The gene *mbtI* (K04781), which encodes salicylic acid synthase in the biosynthesis of the siderophore group nonribosomal peptides pathway, was significantly enriched in both T2 and T3 treatments.

**FIGURE 6:**
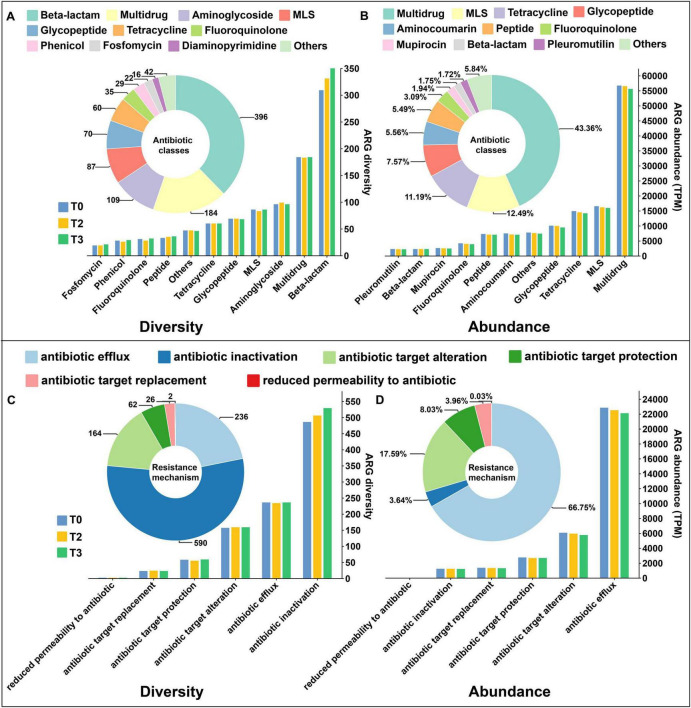
Distribution of ARGs number, classes, and drug resistance mechanism. **(A)** Antibiotic classes based on diversity statistics in unsterilized soil; **(B)** Antibiotic classes based on abundance statistics in unsterilized soil; **(C)** Resistance mechanism based on diversity statistics in unsterilized soil; **(D)** Resistant mechanism based on abundance statistics in unsterilized soil. The pie chart shows the distribution of the number or abundance in overall, and the histogram shows the distribution of the number or abundance of ARGs in each treatment. The data in the figure are four biological repetitions. T0: water; T3: tetramycin 500×.

A total of 1,080 antibiotic resistance genes (ARGs) were detected in the soil. In unsterilized soil, the number of specific resistance genes increased with the concentration of tetramycin (Supplementary Figure 8A), whereas the opposite trend was observed in sterilized soil (Supplementary Figure 8B). The detected genes were classified based on diversity, resulting in 21 classes of antibiotic resistance in unsterilized soil, predominantly represented by Beta-lactam (396), Multidrug (184), and Aminoglycoside (109). Moreover, diversity increased with higher concentrations of tetramycin (Figure 6A). When classified by abundance, the primary antibiotic categories identified included multidrug (43.36%), MLS (12.49%), and tetracycline (11.19%). However, the abundance of these categories decreased as tetramycin concentration increased (Figure 6B). In terms of resistance mechanisms, a majority of detected ARGs were associated with antibiotic inactivation (590 ARGs) and antibiotic efflux (236 ARGs) (Figure 6C). More than 66% of the abundance within the resistance group was attributed to antibiotic efflux genes, while 17.9% was derived from antibiotic target alteration (Figure 6D). The diversity and abundance of resistance mechanisms under tetramycin treatment were consistent with the observed changes in antibiotic categories.

In sterilized soil, the detected ARGs were consistent with those found in unsterilized soil regarding diversity and abundance classification (Supplementary Figures 9A, B). The overall diversity and abundance of these antibiotic classes decreased as tetramycin concentration increased. Additionally, antibiotic inactivation (494 ARGs) and antibiotic efflux (235 ARGs) were identified as the primary resistance mechanisms (Supplementary Figure 9C). The abundance in the resistant group was largely attributed to antibiotic efflux (63.98%) and antibiotic target alteration (18.79%) (Supplementary Figure 9D). Furthermore, the diversity and abundance of these resistance mechanisms were consistent with the observed changes in antibiotic categories.

### 3.6 Effects of different microorganisms on plant disease control and growth promotion

A total of 10 upregulated strains were isolated from the soil treated with tetramycin, comprising 5 bacterial strains and 5 fungal strains; of which, 7 strains demonstrated different antagonistic activities against the pathogen, including BS1, BS2, FS1, FS2, FS3, FS4, FS5 (Supplementary Figure 10A). Additionally, four strains (BS1, BS2, BS5 and FS5) were known to produce siderophores (Supplementary Figure 10B) and five strains (BS2, BS3, FS2, FS4 and FS5) were identified as capable of nitrogen fixation (Supplementary Figure 10C). These different strains were categorized into nine genera at the taxonomic level, BS1 was identified as *Pseudomonas aeruginosa*, BS2 was identified as *Paraburkholderia bannensis*, BS3 was identified as *Variovorax boronicumulans*, BS4 was identified as *Flavobacterium chungangensis*, BS5 was identified as *Microbacterium arabinogalactanolyticum*. FS1 was identified as *Trichoderma atrobrunneum*, FS2 was identified as *Trichoderma atroviride*, FS3 was identified as *Mortierella globalpina*, FS4 was identified as *Penicillium fuscoglaucum*, FS5 was identified as *Cladosporium cycadicola* (Supplementary Figure 11).

**FIGURE 7:**
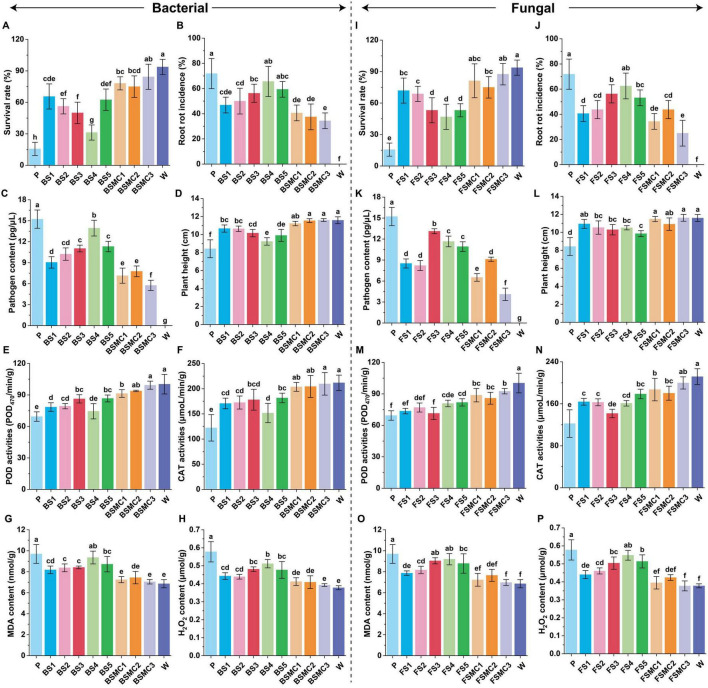
Effects of different microorganisms on plant disease control and growth promotion. **(A)** Survival rate under differential bacterial treatment; **(B)** Root rot incidence under differential bacterial treatment; **(C)** Pathogen content under differential bacterial treatment; **(D)** Plant height under differential bacterial treatment; **(E)** POD activity under differential bacterial treatment; **(F)** CAT activity under differential bacterial treatment; **(G)** MDA content under differential bacterial treatment; **(H)** H_2_O_2_ content under differential bacterial treatment. **(I)** Survival rate under differential fungal treatment; **(J)** Root rot incidence under differential fungal treatment; **(K)** Pathogen content under differential fungal treatment; **(L)** Plant height under differential fungal treatment; **(M)** POD activity under differential fungal treatment; **(N)** CAT activity under differential fungal treatment; **(O)** MDA content under differential fungal treatment; **(P)** H_2_O_2_ content under differential fungal treatment. Different lowercase letters indicate significant differences within groups, *p* < 0.05. The data in the figure are the mean ± standard deviation of six biological replicates. The *p*-values were calculated using one-way analysis of variance and multiple comparisons. P: the addition of *F. solani*; BS1: *Pseudomonas aeruginosa*+*F. solani*; BS2: *Paraburkholderia bannensis*+*F. solani*; BS3: *Variovorax boronicumulans*+*F. solani*; BS4: *Flavobacterium chungangensis*+*F. solani*; BS5: *Microbacterium arabinogalactanolyticum*+*F. solani*; BSMC1: BS1+BS2+*F. solani*; BSMC2: BS3+BS4+BS5+*F. solani*; BSMC3: BS2+BS3+BS4+BS5+*F. solani*. FS1: *Trichoderma atrobrunneum*+*F. solani*; FS2: *Trichoderma atroviride*+*F. solani*; FS3: *Mortierella globalpina*+*F. solani*; FS4: *Penicillium fuscoglaucum*+*F. solani*; FS5: *Cladosporium cycadicola*+*F. solani*; FSMC1: FS1+FS2+FS3+*F. solani*; FSMC2: FS4+FS5+*F. solani*; FSMC3: FS2+FS3+FS4+FS5+*F. solani*. W: water.

The analysis of differential microorganisms and assembly verification revealed that both single and synthetic communities confer disease resistance, induce resistance, and promote plant growth, with the synthetic community exhibiting notably stronger effects than the single strains. Regarding bacterial treatments, the survival rate treated with BSMC1, BSMC2, and BSMC3 increased by 399.99, 380.00, and 439.99%, respectively, compared with that of the control (P) (Figure 7A). The root rot incidence and pathogen content were reduced by 43.48, 47.83, and 52.17% (Figure 7B) and by 53.08, 48.99, and 62.18% (Figure 7C), respectively. There was a notable increase in plant height (Figure 7D) and dry and fresh weights (Supplementary Figures 12A–D) in response to treatments with BSMC1, BSMC2, and BSMC3. Induced resistance results showed that activities of peroxidase (POD) and catalase (CAT) increased by 31.63, 35.18, and 43.06% (Figure 7E), and 66.53, 67.09, and 71.48% (Figure 7F), respectively, over the control. Additionally, malondialdehyde (MDA) and hydrogen peroxide (H_2_O_2_) contents decreased by 25.28, 23.22, and 27.34% (Figure 7G) and 28.67, 29.24, and 31.99% (Figure 7H), respectively. Further analysis showed that these microorganisms had obvious growth-promoting effects (Supplementary Table 3).

In fungal verification, seedlings treated with FSMC1 and FSMC2 exhibited survival rate increases of 420.00% and 459.99%, compared with the control (P) (Figure 7I). Root rot incidence and pathogen content were reduced by 52.10% and 65.22% (Figure 7J) and 57.20% and 73.04% (Figure 7K), respectively. Under pathogen stress, plant height (Figure 7L) and agronomic traits (Supplementary Figures 12E–H) were significantly enhanced. The data supporting induced resistance indicated that relative to those of the control (P), POD and CAT activities increased by 27.90% and 33.36% (Figure 7M), and 53.01%, and 63.40% (Figure 7N), respectively. Moreover, MDA and H_2_O_2_ contents dropped by 25.44% and 28.05% (Figure 7O) and 31.63% and 34.62% (Figure 7P), respectively. It was also confirmed that these microorganisms significantly promotes the growth of *P. notoginseng* (Supplementary Table 4).

### 3.7 Effects of key microorganisms on plant disease control and growth promotion

**FIGURE 8:**
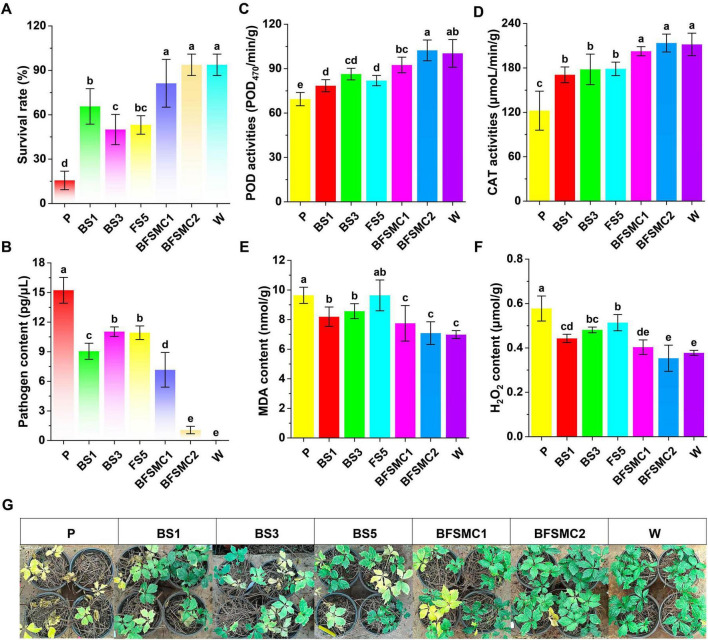
Effects of key microorganisms on plant disease control and growth promotion. **(A)** Survival rate; **(B)** Pathogen content; **(C)** POD activity; **(D)** CAT activity; **(E)** MDA content; **(F)** H_2_O_2_ content. **(G)** Representative images; Different lowercase letters indicate significant differences within groups, *p* < 0.05. The data in the figure are the mean ± standard deviation of six biological replicates. The *p*-values were calculated using one-way analysis of variance and multiple comparisons. P: the addition of *F. solani*; BS1: *Pseudomonas aeruginosa*+*F. solani*; BS3: *Variovorax boronicumulans*+*F. solani*; FS5: *Cladosporium cycadicola*+*F. solani*; BFSMC1: BS1+BS3+*F. solani*; BFSMC2: BS1+BS3+FS5+*F. solani*; W: water.

For the synthetic community by the three key strains, the survival rate with BFSMC1 and BFSMC2 increased by 420.00% and 500.00%, respectively, compared with that of the control (P) (Figure 8A). The root rot incidence and pathogen content decreased by 47.83%, 78.26% (Supplementary Figure 13A) and 52.94%, 93.04% (Figure 8B), respectively. Additionally, the plant height and the agronomic traits were also significantly improved (Supplementary Figures 13B–F). The induced resistance analysis indicated that compared with those of the control (P), the activities of POD and CAT in BFSMC2 increased by 47.41% (Figure 8C) and 74.72% (Figure 8D), respectively, while the contents of MDA and H_2_O_2_ decreased by 27.73% (Figure 8E) and 38.77% (Figure 8F), respectively. The growth-promoting results demonstrated that agronomic traits were also significantly improved (Supplementary Table 5).

## 4 Discussion

### 4.1 The effect of tetramycin in alleviating root rot disease in *P. notoginseng*

Tetramycin is a mixed polyene antibiotic with excellent antimicrobial activity and a unique mode of action (Ma et al., 2018). Previous studies have shown that tetramycin exhibits robust antibacterial activity against a range of pathogens, including *Colletotrichum capsici* and *F. graminearum* (Gao et al., 2018; Shi et al., 2020). This efficacy can be attributed to the ability of tetramycin to disrupt the structure of the cell membrane of pathogens, leading to the rupture of mycelia and the leakage of small molecules and ions. However, the use of tetramycin to mitigate the identified challenges in the cultivation of *P. notoginseng* has not been reported.

Our study showed that 0.3% tetramycin could effectively inhibit the mycelial growth and spore germination of *F. oxysporum*, *F. solani*, and *C. destructans* (Supplementary Figures 1, 2). Meanwhile, it could reduce root rot incidence (Figure 1). We found that the effect of tetramycin was concentration-dependent, this is consistent with the findings of other studies (Xie et al., 2011). The positive effects of appropriate concentrations of tetramycin on the germination rate and growth of *P. notoginseng* may be due to the anti-permeability effect of tetramycin on seeds, which can improve water absorption and induce the synthesis of α-amylase (Ping et al., 2004). High tetramycin concentrations may inhibit plant growth, exert toxic effects on plant cells by disrupting the normal function of plant organelles, and prevent cell division and elongation, thereby inhibiting plant growth (Migliore et al., 2003). Tetramycin treatment of sterilized soil can significantly promote the growth of *P. notoginseng*, indicating that tetramycin has a certain growth-promoting ability.

### 4.2 Microbiome analysis revealed changes in rhizosphere community structure

Microbial communities in the plant rhizosphere are crucial for enhancing plant stress resistance and suppressing pathogens, thereby driving plant defense mechanisms (Philippot et al., 2013). In our study, sequencing of the soil microbiome demonstrated that tetramycin treatment significantly increased the ratio (B/F) of OTUs (Figure 2A), indicating that tetramycin treatment could transform the soil from fungal to bacterial. Diversity analyses revealed that bacterial alpha diversity remained unchanged in unsterilized soil following tetramycin treatment; however, fungal alpha diversity decreased significantly (Supplementary Figures 5A–D), highlighting tetramycin’s stronger impact on fungal diversity. This aligns with previous research findings on wuyiencin (Xie et al., 2023). The stability in bacterial diversity may stem from the robust adaptability of certain bacterial species, while the reduction in fungal diversity might be due to the suppression of sensitive fungal species. Furthermore, PCoA analysis showed distinct bacterial and fungal clusters in the soil (Figures 2B–E), supporting the role of tetramycin in shaping the structure of microbial communities.

Microbial community analysis revealed that Proteobacteria was the predominant group influenced by tetramycin in unsterilized soil (Figure 2F). Proteobacteria play significant roles in balancing plant hormones, enhancing nutrient absorption, and preventing disease invasion (Xu et al., 2018). In terms of fungal community structure, Ascomycota is the principal fungal phylum affected by tetramycin (Figure 2G). Members of Ascomycota are integral to ecosystem functions, including the decomposition of organic matter and nutrient cycling. Ascomycota constitutes a substantial portion of the fungal community, corroborating findings from studies on fungal microbial communities in other crops (Li et al., 2014). A similar community structure was also observed in sterilized soil (Supplementary Figures 6A, B). The findings suggest that there may be a potential link between changes in soil microbiome composition and plant health and disease resistance.

The differential analysis revealed that tetramycin up-regulated bacterial while down-regulating fungal in both unsterilized (Figures 3A, B) and sterilized soil (Figure 3E). Mantel analysis identified *Fusarium* as the primary pathogen (Figure 3K). These findings imply that the observed improvements in seedling survival and the reduction in root rot incidence can be attributed to a decrease in pathogen abundance and an increase in beneficial microorganisms prompted by tetramycin. Additionally, the growth-promoting effects of tetramycin are likely linked to the recruitment of numerous beneficial microorganisms. We isolated and identified 10 differentially abundant microorganisms, with 3 identified as key microorganisms (Supplementary Figure 11). These microorganisms possess unique functional potentials. For example, *Pseudomonas* can inhibit disease onset while promoting plant growth and yield through siderophore production (Shao et al., 2024). *Paraburkholderia* secretes pyrrolnitrin, which inhibits the growth and pathogenicity of fungi (Wang et al., 2023). *Variovorax* promotes the activity of defense-related enzymes to control disease (Yang et al., 2023). *Flavobacterium* offers both growth promotion and disease inhibition (Zheng et al., 2024). *Microbacterium* can boosts host systemic resistance (Berendsen et al., 2018). *Trichoderma*, due to its rapid growth, can dominate nutrients and space near the plant rhizosphere, suppressing pathogenic fungi growth (Panchalingam et al., 2022). *Mortierella* effectively induces resistance against black spot invasion (Ji et al., 2024). *Penicillium* inhibits the colonization of *F. oxysporum* by improving soil properties and increasing beneficial microorganisms (Wang et al., 2022) and *Cladosporium* enhances stress tolerance through secondary metabolites (Chaibub et al., 2016).

The results of community assembly found that stochastic and deterministic processes are critical ecological factors influencing the composition of microbial communities. In unsterilized soil, tetramycin treatment can promote a deterministic process that dominates bacterial community assembly (Figure 4A), In contrast, the assembly of fungal communities transforms from a deterministic to a stochastic process (Figure 4A). Studies have indicated that the assembly process of diseased rhizosphere microbial communities is primarily governed by certainty (Wen et al., 2022). Additionally, the presence of tetracycline may shift bacterial community structure from stochastic process dominance to deterministic process dominance (Zhang et al., 2024; Huang et al., 2024), aligning with the results of this study. The transformation of fungal community assembly into a stochastic process may be attributed to the selective pressures induced by changes in the rhizosphere environment due to tetramycin. In unsterilized soil, both bacterial and fungal community assembly is predominantly stochastic (Figure 4A). This stochastic may stem from the complete removal of original microorganisms in sterilized soil, resulting in weakened competitive bases and selection pressures; consequently, the primary microbial source is recolonization from the external environment (air). Under these conditions, the colonization and diffusion of microorganisms become more stochastic.

### 4.3 Metagenomics reveals the potential disease control mechanism of microorganisms

The PCoA analysis revealed significant separation within the community (Figures 5A, B), aligning with the findings from the microbiome data. The KEGG-based functional enrichment analysis indicated that the treatment of unsterilized soil with tetramycin significantly enriched several key pathways, including the biosynthesis of ansamycins, flagellar assembly, and chemotaxis (Figures 5C, D). Flagella serve as locomotion organs for bacteria, enabling movement toward favorable conditions through chemotaxis (Li et al., 2005). Chemotaxis is crucial for biocontrol bacteria to form biofilms on plant roots, facilitating root colonization and disease prevention (Scharf et al., 2016). Therefore, the enrichment of genes associated with these pathways may enhance microbial colonization and contribute to the transport of essential nutrients, thereby promoting plant growth and improving plant resistance. In sterilized soil, tetramycin treatment significantly enriched the siderophore synthesis pathway (Supplementary Figures 7A, B), with notable upregulation of the gene encoding salicylic acid synthase (mbtI). Microbial siderophores, including salicylic acid, pyochelin, and pyoverdin, can chelate Fe^3 +^ in the soil, reducing its availability to pathogens and inhibiting their growth (Kloepper et al., 1980). Studies have shown that siderophores produced by *Pseudomonas* and *Burkholderia* can induce host ISR (Ho et al., 2021). Additionally, siderophores significantly improve nutrition levels to promote host plant growth (Wang et al., 2024). We also confirmed the capacity of these microorganisms to produce siderophores (Supplementary Figure 10B). Thus, we speculate that the microorganisms recruited by tetramycin may aid in disease prevention and control while promoting plant growth through siderophore production. Interestingly, in the T0 group, where root rot disease was most severe (T0), we observed significant enrichment of numerous metabolic pathways (Figures 5C, D). This phenomenon may result from microorganisms potentially protecting the host plant against pathogens through symbiotic interactions and enhanced metabolic activity. Previous studies have found that kiwifruit endophytes infected by *Botrytis cinerea* can exhibit increased enrichment of secondary metabolite genes associated with antimicrobial activity (Liao et al., 2024). Similarly, long-term continuous cropping of tobacco can recruit functional microorganisms capable of degrading autotoxic substances to alleviate replanting challenges (Xuan et al., 2024). These findings suggest that plants may employ a “cry for help” strategy to recruit beneficial microorganisms to suppress pathogens.

The introduction of tetramycin to unsterilized soil led to an increased prevalence of specific ARGs (Supplementary Figure 8A). Conversely, when tetramycin was introduced to sterilized soil, there was a reduction in the number of specific ARGs (Supplementary Figure 8B). Previous studies have shown a positive correlation between ARG abundance in soil and rising tetracycline concentrations (Li et al., 2019; He et al., 2020). This pattern may result from the high selection pressure induced by tetramycin: resistant strains might become dominant and even generate new ARGs in unsterilized soil. The results of adding tetramycin to sterilized soil demonstrated that tetramycin significantly reduced both the diversity and abundance of ARGs. This indicates that tetramycin helps mitigate the spread and dissemination of ARGs during the re-establishment of microbial communities. Among the resistance mechanisms, antibiotic inactivation is most prevalent, with the main resistance group linked to antibiotic efflux genes (Figures 6C, D and Supplementary Figures 9C, D). Antibiotic efflux is a common defensive strategy used by bacteria to withstand antibacterial pressure, enabling resistance to various antibiotics (Poole, 2005). We hypothesize that the observed increase in these resistance genes may explain the notable recovery capabilities of rhizosphere bacteria following exposure to tetramycin.

Besides, Metagenomic analysis revealed that while tetramycin led to an increase in the diversity of only some ARGs, while their abundance showed a declining trend. This suggests that tetramycin has minimal impact on the diversity and abundance of ARGs, Some studies have also indicated that exposure to biological fungicides can reduce the abundance of ARGs in soil (Sha et al., 2022). Furthermore, it has been found that biological pesticides can degrade rapidly, thereby reducing residue levels and benefiting soil health (Shao and Zhang, 2017; Jiang et al., 2024). These findings provide strong support for the environmentally friendly effects of biological fungicides and demonstrate the high safety of tetramycin for ecosystems. However, specific residue levels and resistance genes still require further testing and verification.

### 4.4 Functional verification of differential microorganisms

Through our omics analysis, we have proposed the roles and relationships of various microorganisms. However, the protective mechanisms and functional roles of these potentially beneficial groups require further validation. We successfully isolated both antagonistic and non-antagonistic strains, confirming their disease control and growth-promoting potential (Figure 7 and Supplementary Tables 3, 4), which aligns with our correlation analysis results. Moreover, the assembled microbial communities demonstrated significantly higher efficacy than individual strains (Figure 7), corroborating previous research findings (Hu et al., 2016). Notably, the antagonistic group (BSMC1 and FSMC1) showed a significant effect in controlling root rot disease (Figures 7B, J), likely due to the bioactive substances produced by specific strains that inhibit pathogens. The non-antagonistic group (BSMC2) also provided effective disease control (Figure 7B). Additionally, the control efficacy of *Trichoderma* (FSMC3) was significantly enhanced when combined with the low-antagonistic fungal group (Figure 7J). Prior studies have suggested that removing specific strains from SynCom can lead directly to its collapse (Niu et al., 2017). Furthermore, we discovered that these distinct microorganisms and synthetic communities significantly activated plant ISR, with the community comprising *Variovorax*, *Microbacterium*, and *Flavobacterium* (BSMC2) having a more profound impact on activating plant ISR compared to individual strains (Figures 7C–F). This finding explains why non-antagonistic bacteria and their assembled communities (BSMC2) can enhance plant disease resistance. Research has suggested that interactions between particular strains and low-abundance strains can synergistically activate ISR to protect plants (Li et al., 2021). Additionally, some rhizosphere bacteria can stimulate the plant jasmonic acid synthesis pathway to produce resistant enzymes that prevent pathogen infection (Saravanakumar et al., 2018). Assembled SynComs can also modulate plant rhizosphere immune responses by influencing the expression of immune-related genes (Ma et al., 2021). These findings highlight the importance of low-abundance strains and induced resistance in promoting plant health and growth. Additionally, the effect of bacteria in activating plant ISR is substantially greater than that of fungi, indicating that bacteria primarily rely on activating plant ISR to combat diseases, whereas fungi primarily rely on direct bacteriostasis.

Furthermore, studies have demonstrated that the cross-kingdom synthetic community (BFSMC2) exhibits robust disease control and growth-promoting capabilities (Figure 8 and Supplementary Table 5). Additionally, research indicates that cross-kingdom synthetic communities are more effective for disease management than the application of fungal or bacterial SynComs in isolation (Zhou et al., 2022). Based on functional experiments, we hypothesize that *Pseudomonas* may produce antagonistic substances and secrete siderophores, thereby directly inhibiting pathogen growth and promoting plant development within the BFSMC2 treatment. On one hand, *Variovorax* could stimulate plant-induced resistance to indirectly suppress pathogen growth; on the other hand, it enhances the nutritional status of plants through nitrogen fixation. *Cladosporium* can generate a significant quantity of spores and siderophores, which allows it to compete with pathogens for resources and further support plant growth. This cross-kingdom synthetic community assembly compensates for functional deficiencies among strains.

By mapping the tetramycin-microorganisms-host association diagram, this study describes in detail the connection between tetramycin and Panax notoginseng diseases (Graphical Abstract). The current research results prove that tetramycin can indirectly recruit microorganisms for disease prevention and to control and promote plant growth, but the pathway of tetramycin attracting beneficial microorganisms under biotic stress, and the signal pathway of disease resistance caused by Cross-kingdom synthetic community needs to be further explored.

## 5 Conclusion

This study presents a novel strategy utilizing tetramycin to alleviate root rot disease in *P. notoginseng*. Our findings indicate that tetramycin can directly inhibit the growth of pathogens. Furthermore, it alters the microbial community structure and potential functions, indirectly recruiting beneficial microorganisms and enriching functional pathways. Additionally, we demonstrated that microorganisms indirectly recruited by tetramycin exhibit strong disease resistance and growth-promoting functions. We confirmed that certain non-antagonistic bacteria can be assembled to mitigate disease interference by activating or enhancing ISR. Furthermore, we found that disease reduction by bacteria primarily relies on the activation of plant ISR, whereas fungi predominantly depend on direct bacteriostasis. The cross-kingdom synthetic community also achieves comprehensive disease control and growth promotion through various mechanisms. These results suggest that the application of tetramycin effectively restores the balance of soil microecology and activates the feedback loop between plants and their associated microorganisms.

## Data Availability

The original contributions presented in this study are included in this article/Supplementary material, further inquiries can be directed to the corresponding authors.

## Footnotes

1
https://github.com/asa12138/reporterscore
