# Supplementary material for: Beneficial soil microbiome profiles assembled using tetramycin to alleviate root rot disease in Panax notoginseng
Source: Front Microbiol. 2025 Apr 29;16:1571684. doi: 10.3389/fmicb.2025.1571684 (PMC12069275; doi:10.3389/fmicb.2025.1571684)
Supplement: Supplementary file 1 [file Table_1.docx]

**Supplementary Material**

**Table S1 Strain composition and addition form for each treatment in the functional verification of strains.**

| Disease prevention verification | | | Growth promotion verification | |
| --- | --- | --- | --- | --- |
| Treatment | Composition | Addition form | Treatment | Addition form |
| P | Fusarium solani | Add only *F. solani* | - | - |
| BS1 | *Pseudomonas aeruginosa* (BS1) | First, the strains to be verified were added, and then *F. solani* was added 24 hours later. | BS1-CK | Only the strain to be verified was added |
| BS2 | *Paraburkholderia bannensis* (BS2) |  | BS2-CK |  |
| BS3 | *Variovorax boronicumulans* (BS3) |  | BS3-CK |  |
| BS4 | *Flavobacterium chungangensis* (BS4) |  | BS4-CK |  |
| BS5 | *Microbacterium*  *arabinogalactanolyticum* (BS5) |  | BS5-CK |  |
| BSMC1 | *Pseudomonas aeruginosa* (BS1) *Paraburkholderia bannensis* (BS2) |  | BSMC1-CK |  |
| BSMC2 | *Variovorax boronicumulans* (BS3) *Flavobacterium chungangensis* (BS4) *Microbacterium*  *arabinogalactanolyticum* (BS5) |  | BSMC2-CK |  |
| BSMC3 | *Paraburkholderia bannensis* (BS2) *Variovorax boronicumulans* (BS3) *Flavobacterium chungangensis* (BS4) *Microbacterium*  *arabinogalactanolyticum* (BS5) |  | BSMC3-CK |  |
| FS1 | *Trichoderma atrobrunneum* (FS1) |  | FS1-CK |  |
| FS2 | *Trichoderma atroviride* (FS2) |  | FS2-CK |  |
| FS3 | *Mortierella globalpina* (FS3) |  | FS3-CK |  |
| FS4 | *Penicillium fuscoglaucum* (FS4) |  | FS4-CK |  |
| FS5 | *Cladosporium cycadicola* (FS5) |  | FS5-CK |  |
| FSMC1 | *Trichoderma atrobrunneum* (FS1) *Trichoderma atroviride* (FS2) *Mortierella globalpina* (FS3) |  | FSMC1-CK |  |
| FSMC2 | *Penicillium fuscoglaucum* (FS4) *Cladosporium cycadicola* (FS5) |  | FSMC2-CK |  |
| FSMC3 | *Trichoderma atroviride* (FS2) *Penicillium fuscoglaucum* (FS4) *Cladosporium cycadicola* (FS5) |  | FSMC3-CK |  |
| BFSMC1 | *Pseudomonas aeruginosa* (BS1) *Variovorax boronicumulans* (BS3) |  | BFSMC1-CK |  |
| BFSMC2 | *Pseudomonas aeruginosa* (BS1) *Variovorax boronicumulans* (BS3) *Cladosporium cycadicola* (FS5) |  | BFSMC2-CK |  |
| W | Sterile water | Add only sterile water | W | Add only sterile water |


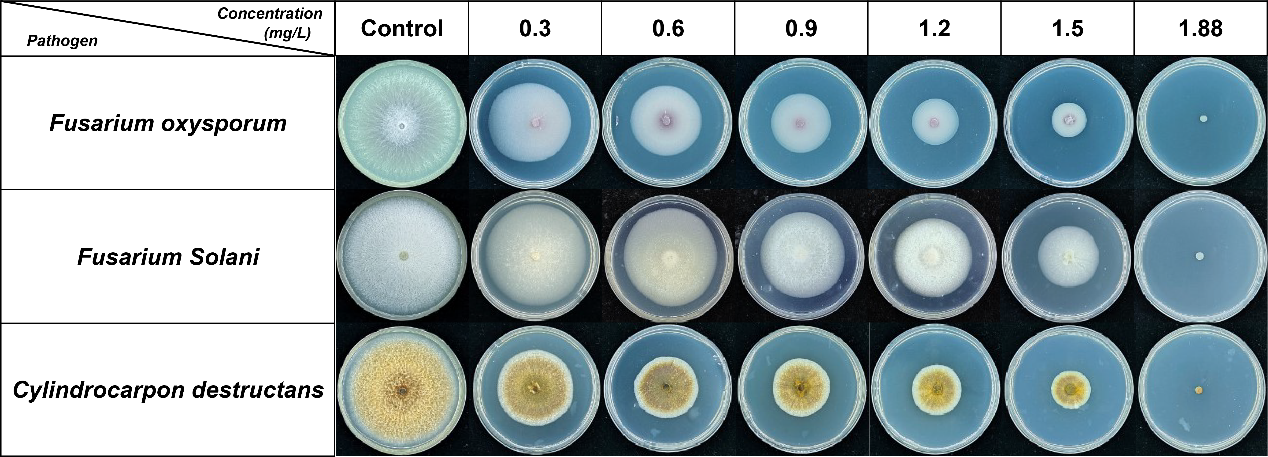


**Figure S1 Inhibitory effects of tetramycin on the mycelial growth of three root rot pathogens of *P. notoginseng***


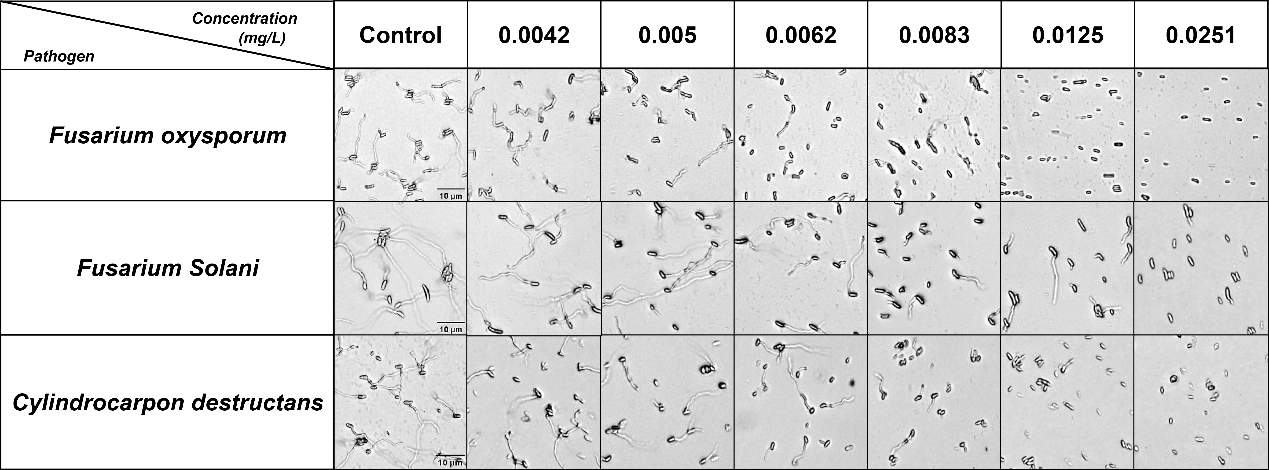


**Figure S2 Inhibitory effects of tetramycin on the spore germination of three root rot pathogens of *P. notoginseng***

**Table S2 Effects of tetramycin on the mycelial growth and spore germination of three root rot pathogens of *P. notoginseng***

| Pathogens tested |  | Concentration | Inhibition rate | EC_50_ | Coefficient correlation |
| --- | --- | --- | --- | --- | --- |
|  |  | (mg/L) | (%) |  |  |
| *Fusarium oxysporum* | Mycelial growth | 0.3 | (35.39 ± 0.94)e | 0.6 | 0.9608 |
|  |  | 0.6 | (46.09 ± 0.36)d |  |  |
|  |  | 0.9 | (54.94 ± 0.62)c |  |  |
|  |  | 1.2 | (68.72 ± 1.28)b |  |  |
|  |  | 1.5 | (77.98 ± 0.36)a |  |  |
| *Fusarium solani* |  | 0.3 | (22.63 ± 0.94)e | 1.52 | 0.9501 |
|  |  | 0.6 | (32.92 ± 0.94)d |  |  |
|  |  | 0.9 | (36.63 ± 0.36)c |  |  |
|  |  | 1.2 | (41.36 ± 0.62)b |  |  |
|  |  | 1.5 | (55.56 ± 0.62)a |  |  |
| *Cylindrocarpon destructans* |  | 0.3 | (41.56 ± 0.94)e | 0.52 | 0.972 |
|  |  | 0.6 | (50.62 ± 0.62)d |  |  |
|  |  | 0.9 | (59.47 ± 0.36)c |  |  |
|  |  | 1.2 | (63.79 ± 1.28)b |  |  |
|  |  | 1.5 | (73.66 ± 0.36)a |  |  |
| *Fusarium oxysporum* | Spore germination | 0.0042 | (13.26 ± 8.75)f | 0.0064 | 0.9687 |
|  |  | 0.005 | (35.12 ± 7.62)e |  |  |
|  |  | 0.0062 | (51.43 ± 11.10)d |  |  |
|  |  | 0.0083 | (59.12 ± 12.74)c |  |  |
|  |  | 0.0125 | (92.56 ± 6.09)b |  |  |
|  |  | 0.0251 | (100.00 ± 0.00)a |  |  |
| *Fusarium solani* |  | 0.0042 | (2.75 ± 2.67)d | 0.0122 | 0.9517 |
|  |  | 0.005 | (5.14 ± 3.86)d |  |  |
|  |  | 0.0062 | (14.66 ± 7.76)c |  |  |
|  |  | 0.0083 | (12.36 ± 5.95)c |  |  |
|  |  | 0.0125 | (28.28 ± 6.84)b |  |  |
|  |  | 0.0251 | (96.94 ± 4.23)a |  |  |
| *Cylindrocarpon destructans* |  | 0.0042 | (2.05 ± 4.64)d | 0.008 | 0.9762 |
|  |  | 0.005 | (24.63 ± 20.54)c |  |  |
|  |  | 0.0062 | (30.87 ± 18.23)c |  |  |
|  |  | 0.0083 | (43.64 ± 16.96)b |  |  |
|  |  | 0.0125 | (92.49 ± 12.22)a |  |  |
|  |  | 0.0251 | (99.59 ± 0.79)a |  |  |

Different lowercase letters indicate significant differences between groups, *p* < 0.05. The data in the table are the mean ± standard deviation of six biological replicates. The *p*-values were calculated using one-way analysis of variance and multiple comparisons.


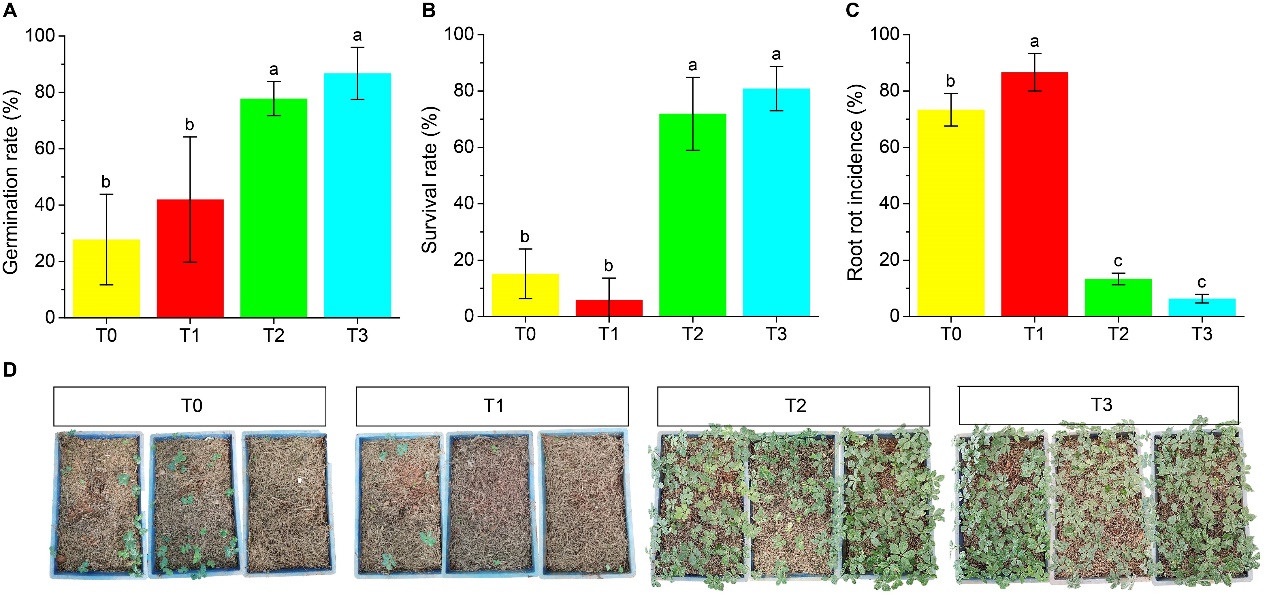


**Figure S3 Effect of tetramycin on alleviating root rot of *P. notoginseng***

(A) Effects of tetramycin on the germination rate of *P. notoginseng.* (B) Effects of tetramycin on the survival rate of *P. notoginseng.* (C) Effects of tetramycin on the disease rate of *P. notoginseng.* Different lowercase letters indicate significant differences within groups, (D) Representative images*. p* < 0.05. The data in the figure are the mean ± standard deviation of three biological replicates. The *p*-values were calculated using one-way analysis of variance and multiple comparisons. T0: water; T1: tetramycin 2000×; T2: 1000×; T3: tetramycin 500×.

***
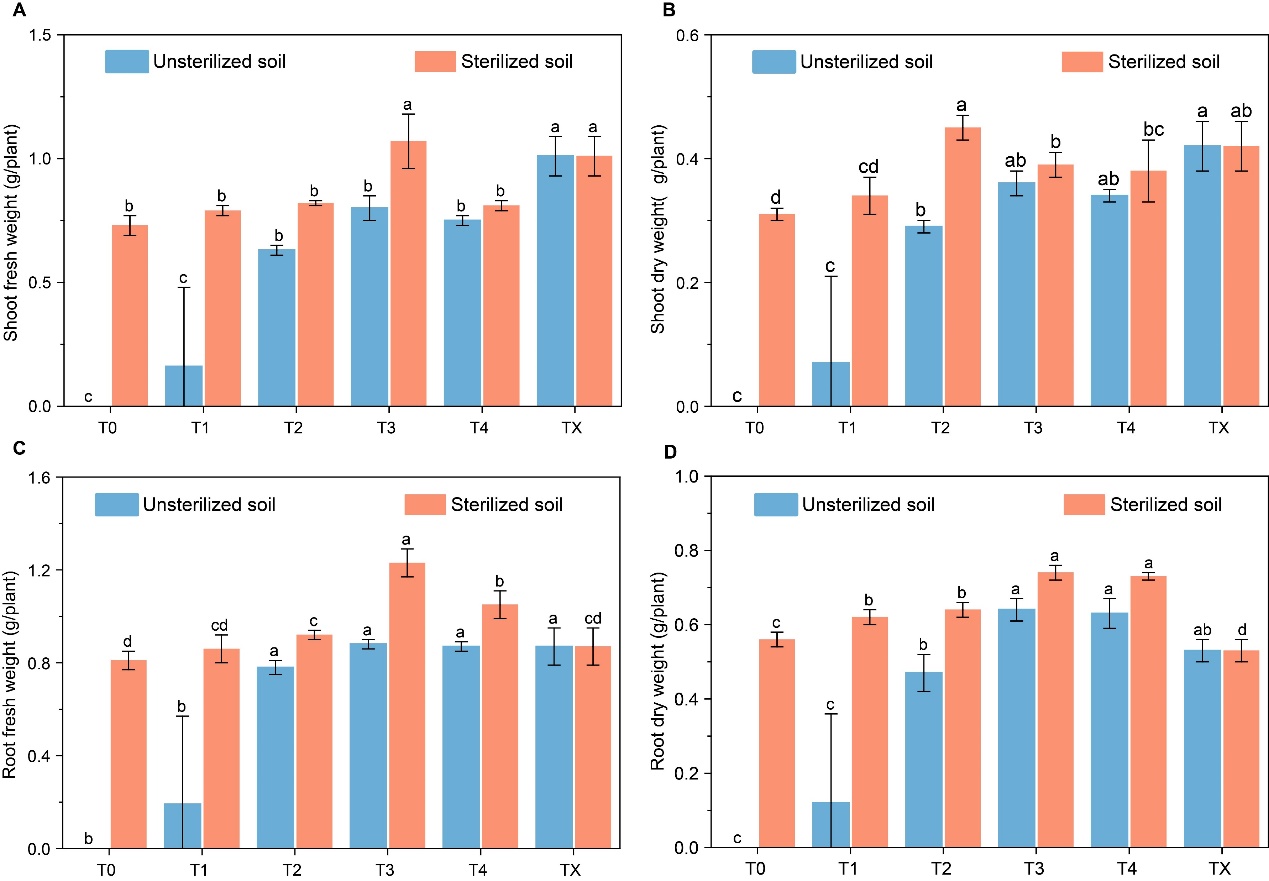
***

**Figure S4 Effects of tetramycin on the agronomic traits of *P. notoginseng***

(A) Effects of tetramycin on the shoot fresh weight of *P. notoginseng.* (B) Effects of tetramycin on the shoot dry weight of *P. notoginseng.* (C) Effects of tetramycin on the root fresh weight of *P. notoginseng.* (D) Effects of tetramycin on the root dry weight of *P. notoginseng.* Different lowercase letters indicate significant differences within groups, *p* < 0.05. The data in the figure are the mean ± standard deviation of four biological replicates. The *p*-values were calculated using one-way analysis of variance and multiple comparisons. T0: water; T1: tetramycin 2000×; T2: 1000×; T3: tetramycin 500×; T4: tetramycin 250×; TX: new soil under the forest.


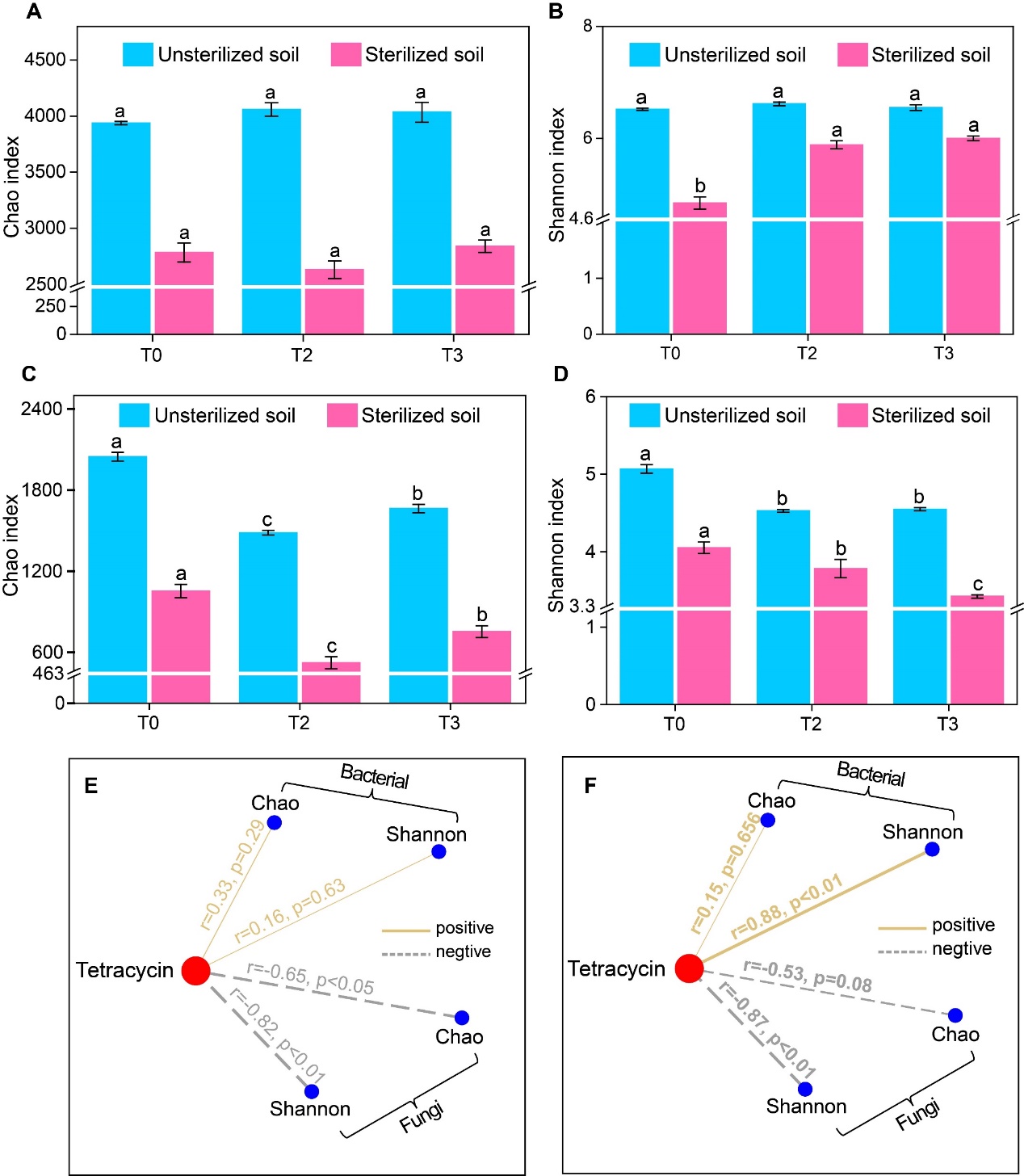


**Figure S5 Effect of tetramycin on microbial community diversity**

(A–B) Chao and Shannon indices of bacteria community. (C–D) Chao and Shannon indices of fungi community. (E) Correlation analysis between α diversity and tetramycin in unsterilized. (F) correlation analysis between α diversity and tetramycin in sterilized. Different lowercase letters indicate significant differences within groups, *p* < 0.05. The data in the figure are the mean ± standard deviation of four biological replicates. The *p*-values were calculated using one-way analysis of variance and multiple comparisons. T0: water; T2: 1000×; T3: tetramycin 500×.


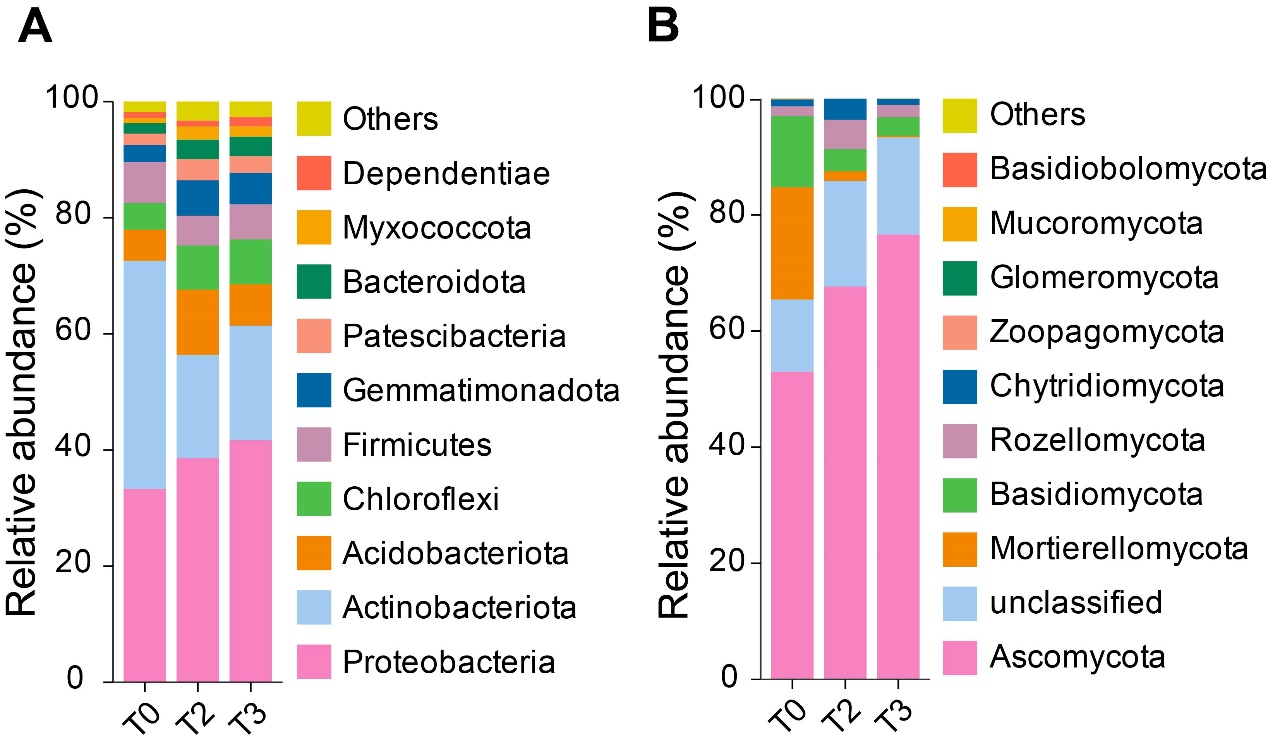


**Figure S6 Effect of tetramycin on microbial community composition**

(A) Species composition analysis based on phylum level in sterilized soil. (B) Species composition analysis based on phylum level in sterilized soil. T0: water; T2: 1000×; T3: tetramycin 500×.

**
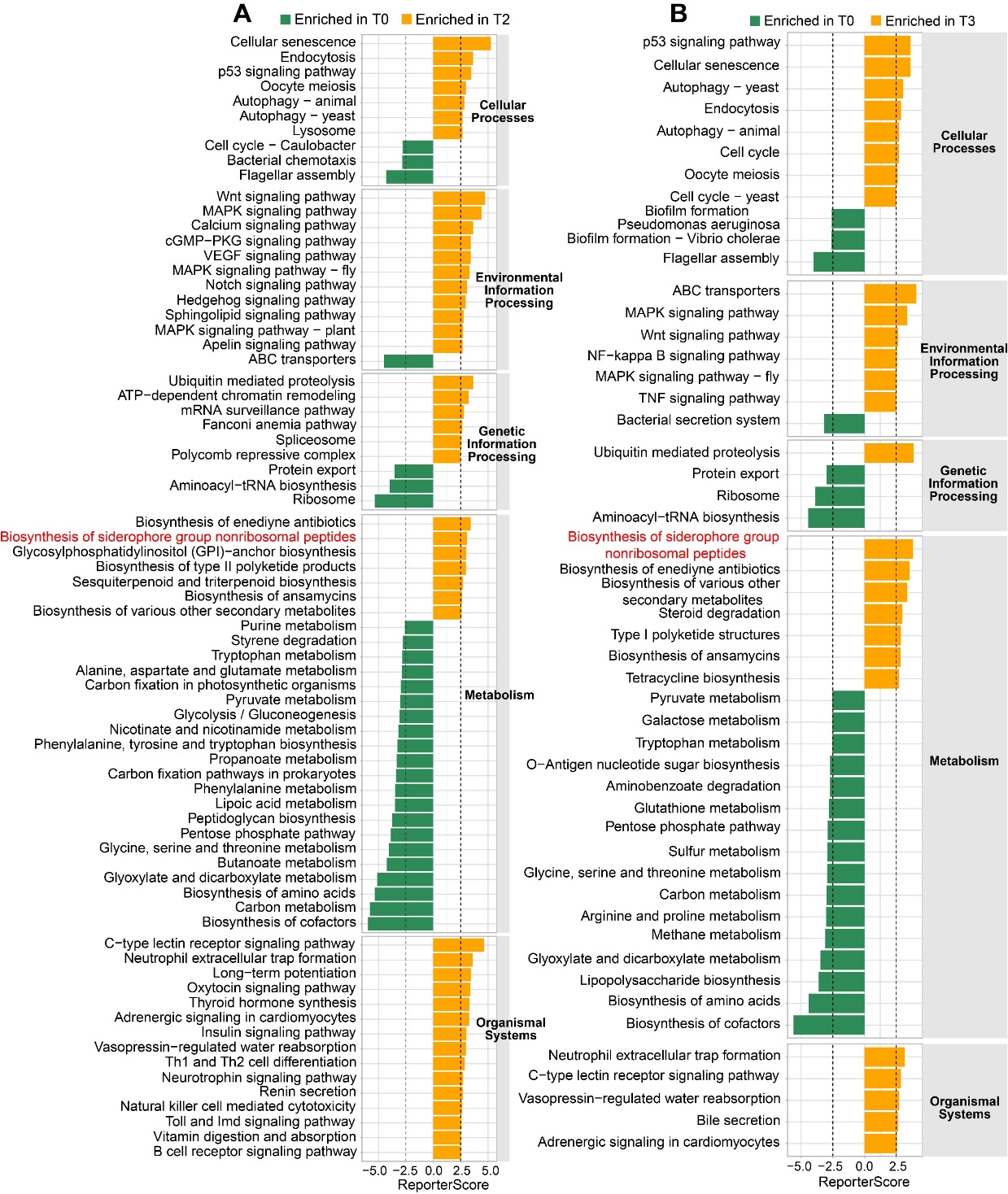
**

**Figure S7 Generalized reporter score-based (GRSA) enrichment analysis**

(A) KEGG pathway enrichment analysis under T2 treatment in sterilized soil. (B) KEGG pathway enrichment analysis under T3 treatment in sterilized soil. The score threshold of the report was 2.5, and the corresponding confidence level was approximately 0.995. The human disease pathway was removed, and the significantly enriched pathways in T0 are displayed in green. The significantly enriched pathways in T2 or T3 are shown as orange. The data in the figure are four biological repetitions. T0: water; T2: 1000×; T3: tetramycin 500×.


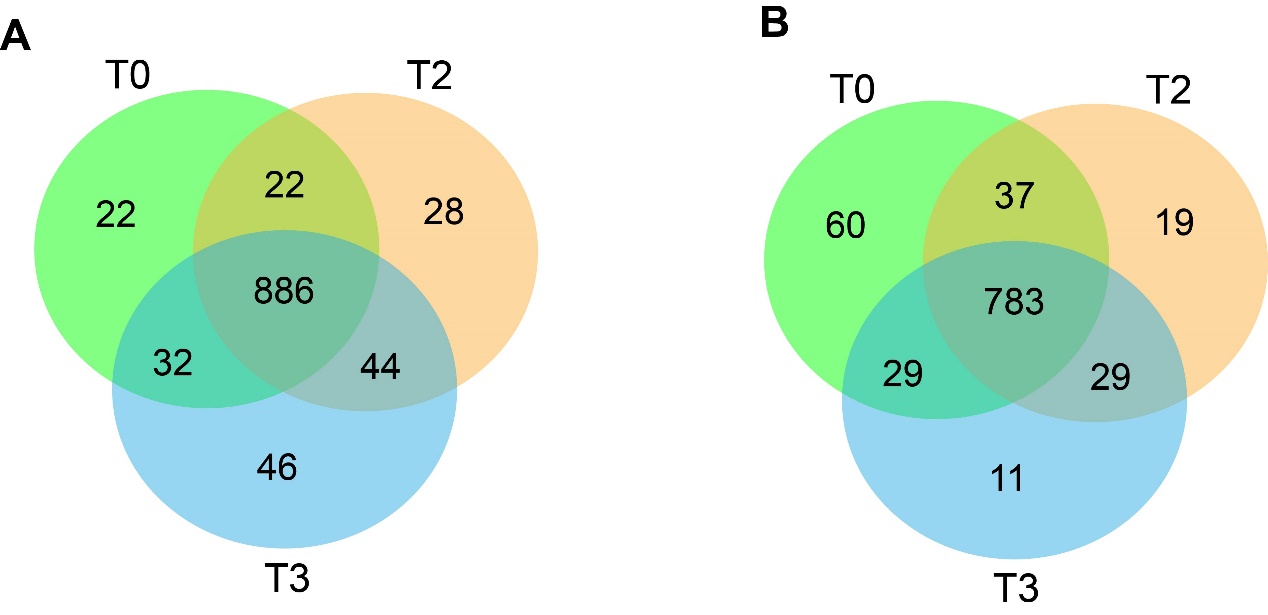


**Figure S8** **Distribution of ARGs number**

(A) Distribution of ARGs number in unsterilized soil; (B) Distribution of ARGs number in sterilized soil. The data in the figure are the mean ± standard deviation of four biological replicates. T0: water; T2: 1000×; T3: tetramycin 500×.


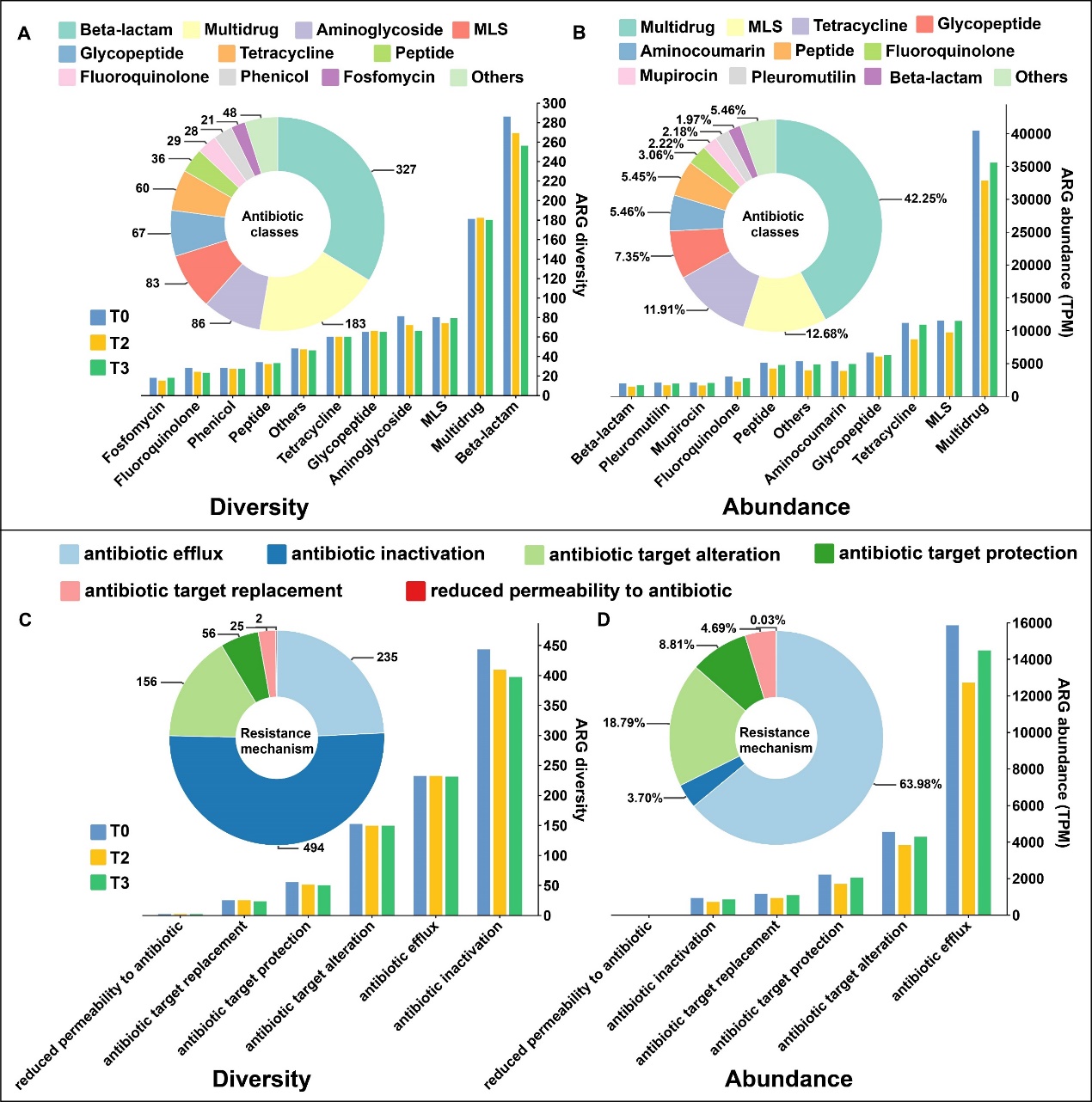


**Figure S9 Distribution of ARGs number, classes, and drug resistance mechanism**

(A) Antibiotic classes based on diversity statistics in sterilized soil; (B) Antibiotic classes based on abundance statistics in sterilized soil; (C) Resistance mechanism based on diversity statistics in sterilized soil; (D) Resistant mechanism based on abundance statistics in sterilized soil. The pie chart shows the distribution of the number or abundance in overall, and the histogram shows the distribution of the number or abundance of arg in each treatment. The data in the figure are four biological repetitions. T0: water; T3: tetramycin 500×.


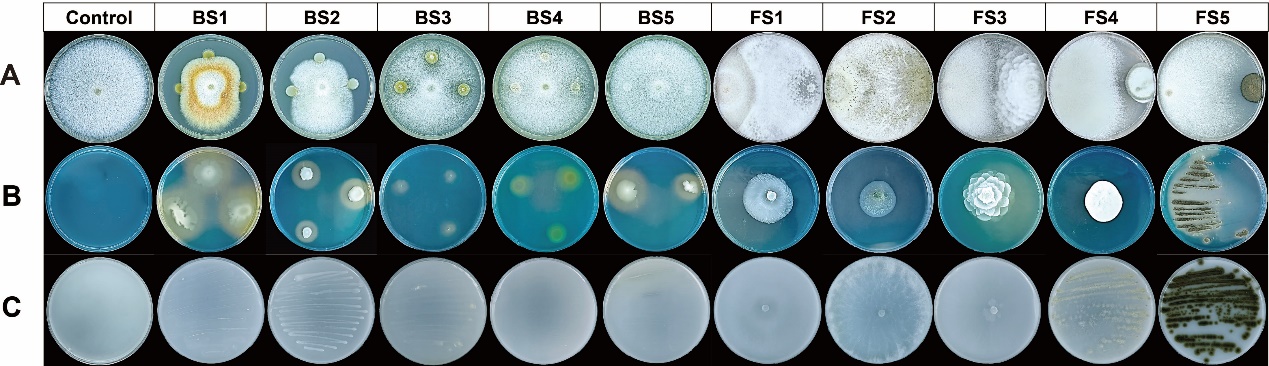


**Figure S10 Microbial isolation and functional identification**

(A) Confrontation culture；(B) Screening of siderophore-producing strains；(C) Screening of nitrogen-fixing strains. BS1: *Pseudomonas aeruginosa*; BS2: *Paraburkholderia bannensis*; BS3: *Variovorax boronicumulans*; BS4: *Flavobacterium chungangensis*; BS5: *Microbacterium arabinogalactanolyticum*; FS1: *Trichoderma atrobrunneum*; FS2: *Trichoderma atroviride*; FS3: *Mortierella globalpina*; FS4: *Penicillium fuscoglaucum*; FS5: *Cladosporium cycadicola*.


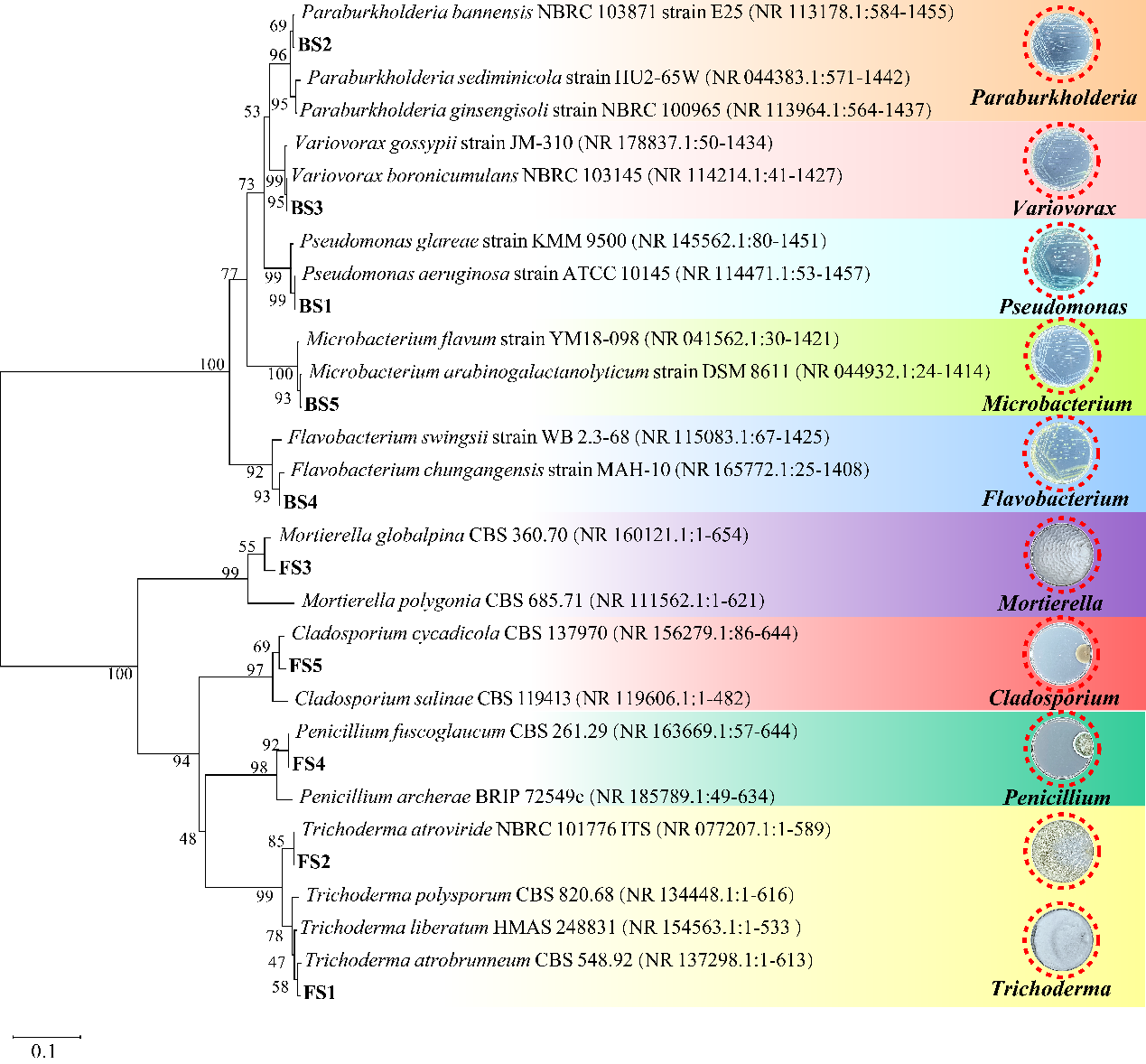


**Figure S11 Identification of differential microbial groups**

The NJ phylogenetic tree was constructed based on the 16S rRNA or ITS gene, and the colony morphological characteristics of each strain on the medium were shown on the right side. BS1: *Pseudomonas aeruginosa*; BS2: *Paraburkholderia bannensis*; BS3: *Variovorax boronicumulans*; BS4: *Flavobacterium chungangensis*; BS5: *Microbacterium arabinogalactanolyticum*; FS1: *Trichoderma atrobrunneum*; FS2: *Trichoderma atroviride*; FS3: *Mortierella globalpina*; FS4: *Penicillium fuscoglaucum*; FS5: *Cladosporium cycadicola*.
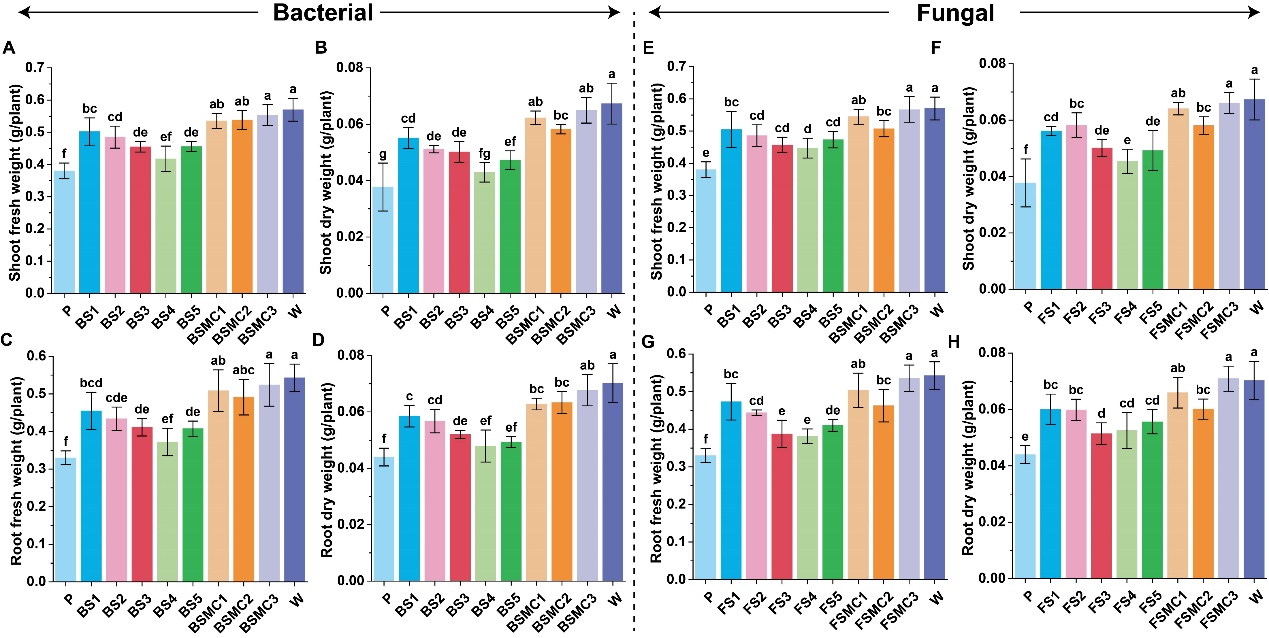


**Figure S12 Effects of different microorganisms on plant disease control and growth promotion**

(A) Shoot fresh weight under differential bacterial treatment; (B) Shoot dry weight under differential bacterial treatment; (C) Root fresh weight under differential bacterial treatment; (D) Root dry weight under differential bacterial treatment; (E) Shoot fresh weight under differential fungal treatment; (F) Shoot dry weight under differential fungal treatment; (G) Root fresh weight under differential fungal treatment; (H) Root dry weight under differential fungal treatment; *p* < 0.05. The data in the figure are the mean ± standard deviation of six biological replicates. The *p*-values were calculated using one-way analysis of variance and multiple comparisons. P: the addition of *F. solani*; BS1: *Pseudomonas aeruginosa*+*F. solani*; BS2: *Paraburkholderia bannensis*+*F. solani*; BS3: *Variovorax boronicumulans*+*F. solani*; BS4: *Flavobacterium chungangensis*+*F. solani*; BS5: *Microbacterium arabinogalactanolyticum*+*F. solani*; BSMC1: BS1+BS2+*F. solani*; BSMC2: BS3+BS4+BS5+*F. solani*; BSMC3: BS2+BS3+BS4+BS5+*F. solani*. FS1: *Trichoderma atrobrunneum*+*F. solani*; FS2: *Trichoderma atroviride*+*F. solani*; FS3: *Mortierella globalpina*+*F. solani*; FS4: *Penicillium fuscoglaucum*+*F. solani*; FS5: *Cladosporium cycadicola*+*F. solani*; FSMC1: FS1+FS2+FS3+*F. solani*; FSMC2: FS4+FS5+*F. solani*; FSMC3: FS2+FS3+FS4+FS5+*F. solani*. W: water.

**Table S3 Growth-promoting effect of different bacteria on *P. notoginseng***

| Treatment | Plant height (cm/plant) | Shoot fresh weight (g/plant) | Shoot dry weight (g/plant) | Root fresh weight (g/plant) | Root dry weight (g/plant) |
| --- | --- | --- | --- | --- | --- |
| BS1-CK | 12.56 ± 0.16 bcd | 0.69 ± 0.03 bc | 0.08 ± 0.00 ab | 0.69 ± 0.02 bc | 0.08 ± 0.01 ab |
| BS2-CK | 12.48 ± 0.37 cd | 0.69 ± 0.02 bc | 0.08 ± 0.00 bc | 0.67 ± 0.03 cd | 0.08 ± 0.01 ab |
| BS3-CK | 12.33 ± 0.30 d | 0.65 ± 0.03 cd | 0.07 ± 0.00 bc | 0.65 ± 0.02 cd | 0.08 ± 0.00 abc |
| BS4-CK | 11.63 ± 0.12 e | 0.61 ± 0.04 de | 0.07 ± 0.00 cd | 0.59 ± 0.05 e | 0.07 ± 0.00 bc |
| BS5-CK | 12.25 ± 0.21 d | 0.64 ± 0.03 cd | 0.07 ± 0.00 bc | 0.62 ± 0.03 de | 0.08 ± 0.00 abc |
| BSMC1-CK | 12.81 ± 0.29 bc | 0.73 ± 0.03 ab | 0.08 ± 0.00 a | 0.74 ± 0.03 a | 0.08 ± 0.00 a |
| BSMC2-CK | 12.95 ± 0.14 ab | 0.76 ± 0.03 a | 0.08 ± 0.00 a | 0.72 ± 0.03 ab | 0.08 ± 0.01 a |
| BSMC3-CK | 13.25 ± 0.27 a | 0.78 ± 0.06 a | 0.08 ± 0.00 a | 0.76 ± 0.03 a | 0.09 ± 0.01 a |
| W | 11.59 ± 0.38 e | 0.57 ± 0.04 e | 0.07 ± 0.01 d | 0.54 ± 0.04 f | 0.07 ± 0.01 c |

Different lowercase letters indicate significant differences within groups, *p* < 0.05. The data in the figure are the mean ± standard deviation of six biological replicates. The *p*-values were calculated using one-way analysis of variance and multiple comparisons. BS1-CK: inoculate only with *Pseudomonas aeruginosa*; BS2-CK: inoculate only with *Paraburkholderia bannensis*; BS3-CK: inoculate only with *Variovorax boronicumulans*; BS4-CK: inoculate only with *Flavobacterium chungangensis*; BS5-CK: inoculate only with *Microbacterium arabinogalactanolyticum*; BSMC1-CK: inoculate only with BS1+BS2; BSMC2-CK: inoculate only with BS3+BS4+BS5; BSMC3-CK: inoculate only with BS2+BS3+BS4+BS5; W: inoculate only with sterile water.

**Table S4 Growth-promoting effect of different fungal on *P. notoginseng***

| Treatment | Plant height (cm/plant) | Shoot fresh weight (g/plant) | Shoot dry weight (g/plant) | Root fresh weight (g/plant) | Root dry weight (g/plant) |
| --- | --- | --- | --- | --- | --- |
| FS1-CK | 12.72 ± 0.22 bc | 0.73 ± 0.04 b | 0.08 ± 0.01 bc | 0.70 ± 0.03 bc | 0.08 ± 0.01 ab |
| FS2-CK | 12.67 ± 0.38 bc | 0.70 ± 0.04 bc | 0.08 ± 0.00 cd | 0.67 ± 0.02 bcd | 0.08 ± 0.00 b |
| FS3-CK | 11.76 ± 0.55 de | 0.64 ± 0.03 d | 0.07 ± 0.00 de | 0.60 ± 0.04 e | 0.07 ± 0.00 bc |
| FS4-CK | 11.97 ± 0.37 de | 0.64 ± 0.03 cd | 0.07 ± 0.00 cde | 0.62 ± 0.04 de | 0.08 ± 0.01 bc |
| FS5-CK | 12.24 ± 0.48 cd | 0.66 ± 0.02 cd | 0.07 ± 0.00 cd | 0.66 ± 0.03 cd | 0.08 ± 0.00 bc |
| FSMC1-CK | 13.14 ± 0.50 ab | 0.81 ± 0.04 a | 0.08 ± 0.00 ab | 0.79 ± 0.03 a | 0.09 ± 0.00 a |
| FSMC2-CK | 12.63 ± 0.37 bc | 0.74 ± 0.04 b | 0.08 ± 0.00 bc | 0.71 ± 0.03 b | 0.08 ± 0.00 ab |
| FSMC3-CK | 13.33 ± 0.04 a | 0.83 ± 0.05 a | 0.09 ± 0.00 a | 0.77 ± 0.04 a | 0.09 ± 0.00 a |
| W | 11.59 ± 0.38 e | 0.57 ± 0.04 e | 0.07 ± 0.01 e | 0.54 ± 0.04 f | 0.07 ± 0.01 c |

Different lowercase letters indicate significant differences within groups, *p* < 0.05. The data in the figure are the mean ± standard deviation of six biological replicates. The *p*-values were calculated using one-way analysis of variance and multiple comparisons. FS1-CK: inoculate only with *Trichoderma atrobrunneum*; FS2-CK: inoculate only with *Trichoderma atroviride*; FS3-CK: inoculate only with *Mortierella globalpina*; FS4-CK: inoculate only with *Penicillium fuscoglaucum*; FS5-CK: inoculate only with *Cladosporium cycadicola*; FSMC1-CK: inoculate only with FS1+FS2+FS3; FSMC2-CK: inoculate only with FS4+FS5; FSMC3-CK: inoculate only with FS2+FS3+FS4+FS5; W: inoculate only with sterile water.


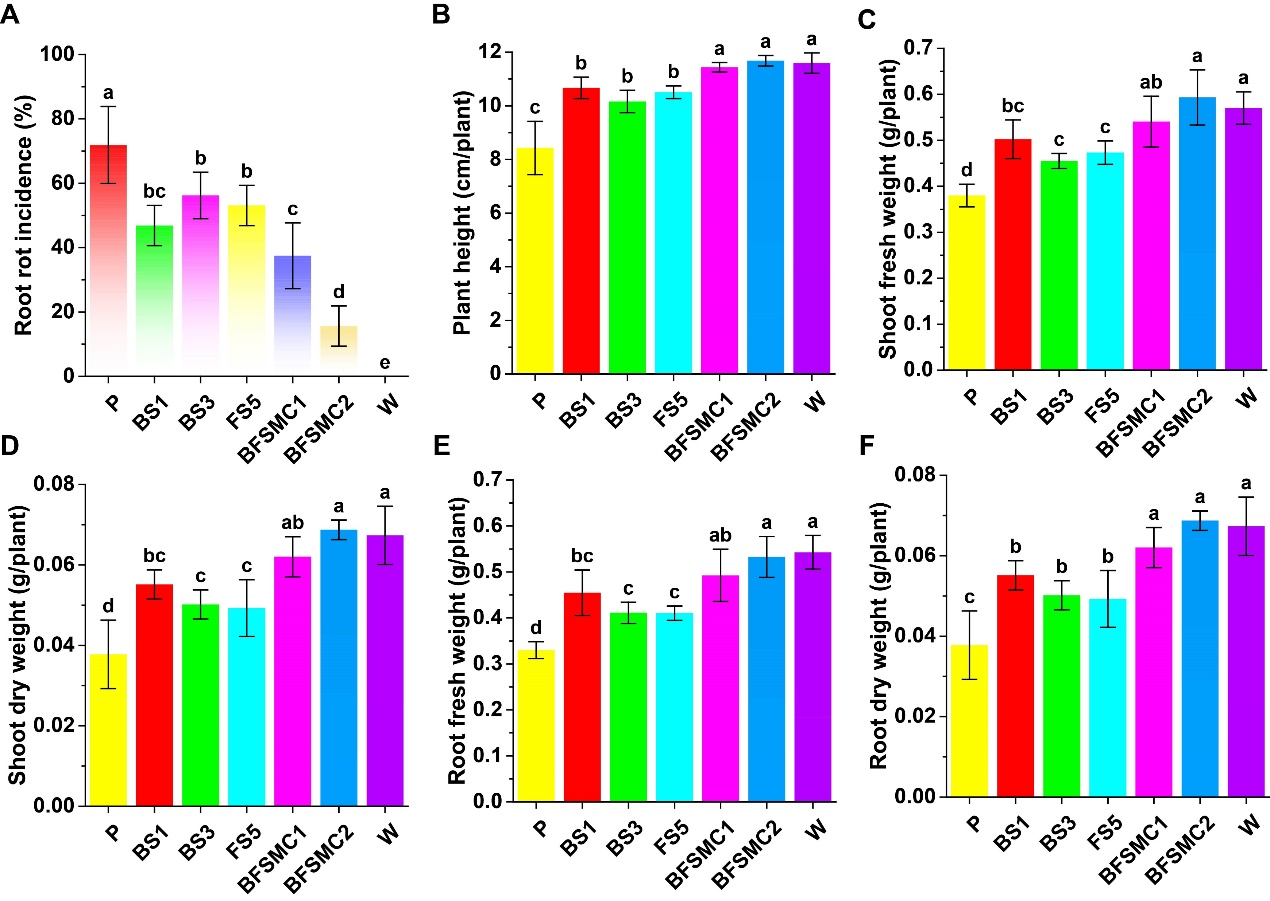


**Figure S13 Effects of key microorganisms on plant disease control and growth promotion**

(A) Root rot incidence; (B) Plant height; (C) Shoot fresh weight; (D) Shoot dry weight; (E) Root fresh weight; (F) Root dry weight. Different lowercase letters indicate significant differences within groups, *p* < 0.05. The data in the figure are the mean ± standard deviation of six biological replicates. The *p*-values were calculated using one-way analysis of variance and multiple comparisons. P: the addition of *F. solani*; BS1: *Pseudomonas*+*F. solani*; BS3: *Variovorax*+*F. solani*; FS5: *Cladosporium*+*F. solani*; BFSMC1: BS1+BS3+*F. solani*; BFSMC2: BS1+BS3+FS5+*F. solani*; W: water.

**Table S5 Growth-promoting effect of key fungal on *P. notoginseng***

| Treatment | Plant height (cm/plant) | Shoot fresh weight (g/plant) | Shoot dry weight (g/plant) | Root fresh weight (g/plant) | Root dry weight (g/plant) |
| --- | --- | --- | --- | --- | --- |
| BS1-CK | 12.56 ± 0.16 bc | 0.69 ± 0.03 c | 0.08 ± 0.00 bc | 0.69 ± 0.02 c | 0.08 ± 0.01 bc |
| BS3-CK | 12.33 ± 0.30 c | 0.65 ± 0.03 c | 0.07 ± 0.00 cd | 0.65 ± 0.02 c | 0.08 ± 0.00 bcd |
| FS5-CK | 12.24 ± 0.48 c | 0.66 ± 0.02 c | 0.07 ± 0.00 cd | 0.66 ± 0.03 c | 0.08 ± 0.00 cd |
| BFSMC1-CK | 13.02 ± 0.28 ab | 0.75 ± 0.05 b | 0.08 ± 0.00 ab | 0.72 ± 0.03 b | 0.08 ± 0.01 ab |
| BFSMC2-CK | 13.52 ± 0.49 a | 0.85 ± 0.04 a | 0.09 ± 0.00 a | 0.81 ± 0.01 a | 0.09 ± 0.01 a |
| W | 11.59 ± 0.38 d | 0.57 ± 0.04 d | 0.07 ± 0.01 d | 0.54 ± 0.04 d | 0.07 ± 0.01 d |

Different lowercase letters indicate significant differences within groups, *p* < 0.05. The data in the figure are the mean ± standard deviation of six biological replicates. The *p*-values were calculated using one-way analysis of variance and multiple comparisons. BS1-CK: inoculate only with *Pseudomonas aeruginosa*; BS3-CK: inoculate only with *Variovorax boronicumulans*; FS5-CK: inoculate only with *Cladosporium cycadicola*; BFSMC1-CK: inoculate only with BS1+BS3; BFSMC2-CK: inoculate only with BS1+BS3+FS5; W: inoculate only with sterile water.
